# Distinct Prion Domain Sequences Ensure Efficient Amyloid Propagation by Promoting Chaperone Binding or Processing *In Vivo*

**DOI:** 10.1371/journal.pgen.1006417

**Published:** 2016-11-04

**Authors:** Christine R. Langlois, Fen Pei, Suzanne S. Sindi, Tricia R. Serio

**Affiliations:** 1 Department of Molecular and Cellular Biology, The University of Arizona, Tucson, Arizona, United States of America; 2 Department of Molecular Biology, Cell Biology and Biochemistry, Brown University, Providence, Rhode Island, United States of America; 3 Applied Mathematics, School of Natural Sciences, University of California, Merced, Merced, California, United States of America; University of Kent, UNITED KINGDOM

## Abstract

Prions are a group of proteins that can adopt a spectrum of metastable conformations *in vivo*. These alternative states change protein function and are self-replicating and transmissible, creating protein-based elements of inheritance and infectivity. Prion conformational flexibility is encoded in the amino acid composition and sequence of the protein, which dictate its ability not only to form an ordered aggregate known as amyloid but also to maintain and transmit this structure *in vivo*. But, while we can effectively predict amyloid propensity *in vitro*, the mechanism by which sequence elements promote prion propagation *in vivo* remains unclear. In yeast, propagation of the [*PSI*^*+*^] prion, the amyloid form of the Sup35 protein, has been linked to an oligopeptide repeat region of the protein. Here, we demonstrate that this region is composed of separable functional elements, the repeats themselves and a repeat proximal region, which are both required for efficient prion propagation. Changes in the numbers of these elements do not alter the physical properties of Sup35 amyloid, but their presence promotes amyloid fragmentation, and therefore maintenance, by molecular chaperones. Rather than acting redundantly, our observations suggest that these sequence elements make complementary contributions to prion propagation, with the repeat proximal region promoting chaperone binding to and the repeats promoting chaperone processing of Sup35 amyloid.

## Author Summary

Protein misfolding and assembly into ordered aggregates known as amyloid has emerged as a novel mechanism for regulation of protein function. In the case of prion proteins, the resulting amyloid is transmissible, creating protein-based elements of infectivity and inheritance. These unusual properties are linked to the amino acid composition and sequence of the protein, which confer both conformational flexibility and persistence *in vivo*, the latter of which occurs through mechanisms that are currently poorly understood. Here, we address this open question by studying a region of the yeast prion Sup35 that has been genetically linked to persistence. We find that this region is composed of two separable elements that are both required for efficient persistence of the amyloid. These elements do not contribute to amyloid stability. Rather, they promote distinct aspects of its functional interactions with molecular chaperones, which are required for efficient conformational self-replication and transmission.

## Introduction

The ability of some proteins to adopt alternative conformations expands the functional range of the proteome by allowing individual proteins to access multiple conformers. For a unique and expanding class of proteins, these conformational transitions are extensive and lead to the assembly of the protein into ordered, linear, β-sheet-rich aggregates, known as amyloid [1]. The amyloid conformation is self-perpetuating, allowing the activity of the alternative conformer to dominate, and in the case of a subset of amyloids known as prions, to become transmissible [2].

These alternative conformational states are often associated with new phenotypes, arising from either the loss of normal functions or the gain of new functions for the proteins [2]. Indeed, amyloids and prions contribute both to normal cellular homeostasis, regulating gene expression, immunity, memory, organelle biogenesis, and ultrastructure [2–6], and to the disruption of this balance through diseases, including neurodegeneration, Type II diabetes, and familial hypertension [1]. But, these biological consequences only arise because the amyloid structure, once it appears, can persist *in vivo*. One component of this persistence, is the ability of the linear amyloid fibers to template the refolding of normal conformers of the same protein into the alternative state through interactions at the ends of the aggregates [1]. This propensity to undergo self-templated conformational replication is an inherent property of each protein, dictated by the composition and sequence of its amino acids, typically in one region of the protein that is predicted to be intrinsically disordered [7]. These regions tend to be rich in glutamine and/or asparagine (Q/N), which promote aggregation via their propensity to form hydrogen-bonding networks [8–10], and depleted for prolines and charged amino acids, which are thought to act as amyloid breakers [11]. As such, the spacing of these latter residues, when present, is an important predictor of amyloidogenicity [11,12].

Beyond the formation of the amyloid structure, the amplification and persistence (i.e. propagation) of these complexes *in vivo* is also absolutely essential to realize their physiological consequences. In contrast to amyloid formation, the molecular basis of amyloid propagation is far less well understood. For example, a computational study aimed at identifying prion proteins in the yeast proteome based on sequence characteristics effectively predicted proteins capable of self-assembling into SDS-resistant aggregates, a hallmark of the amyloid state. However, only a small subset of these aggregation-prone proteins could support inheritance of an amyloid reporter-based phenotype [12], indicating our limited ability to predict the persistence of these complexes *in vivo*.

Nonetheless, some insight into the protein characteristics that support amyloid propagation has been gleaned from extensive analysis of the yeast prion protein Sup35, a translation release factor that can access a heritable amyloid state known as [*PSI*^*+*^] *in vivo* [2]. The Sup35 protein contains a bipartite N-terminal prion-determining domain (PrD) composed of a Q/N-rich region (amino acids 1–41) followed by five and a half imperfect oligopeptide repeats (amino acids 42–97) with the consensus sequence PQGGYGGYN [13]. The Q/N-rich region is required for and confers specificity to the aggregation [14,15], but this region alone is not sufficient to induce or sustain [*PSI*^*+*^] *in vivo* [16,17]. Instead, these activities depend on the oligopeptide repeat region [16–25]. The addition of the first repeat to the Sup35 Q/N-rich region allows the protein to join existing amyloid aggregates composed of full-length Sup35 *in vivo*, but five repeats, in addition to the Q/N-rich region, are required for prion propagation in the absence of full-length Sup35 [16,17]. Consistent with these observations, the addition of the Sup35 oligopeptide repeats converted an aggregation-prone polyglutamine track (Q62) to a heritable prion [16], demonstrating that the essential function of the oligopeptide repeats in prion propagation is transferrable.

The number of Sup35 oligopeptide repeats has also been implicated in the stability of the non-prion [*psi*^-^] state *in vivo*. Deletion of four repeats leads to a decrease in the spontaneous frequency of [*PSI*^*+*^] appearance, while the introduction of two additional repeats leads to an increase in the spontaneous frequency of [*PSI*^*+*^] appearance *in vivo*, with both changes contributing a two-order-of-magnitude effect [21]. Both the repeat deleted and expanded proteins retain the ability to form amyloid *in vitro*, albeit at different rates, suggesting that the contribution of the repeats to prion propagation *in vivo* cannot be explained solely by aggregation propensity [21].

The mammalian prion protein, PrP, also contains five oligopeptide repeats, four with the sequence PHGGGWGQ and a fifth with the sequence PQGGGTWGQ [26,27]. In humans, expansion of the repeats, ranging from a single to twelve additional copies, is associated with neurodegenerative disease [28,29]. Paralleling the observations of [*PSI*^*+*^] appearance in yeast, the age of onset of neurodegenerative disease in humans correlates inversely with the number of PrP repeats [30–32]. Intriguingly, PrP repeats can substitute for the essential function of Sup35 oligopeptide repeats in the maintenance of [*PSI*^*+*^] in yeast, indicating functional overlap [17,33,34].

Despite this functional significance and evolutionary conservation [35], the exact mechanism by which Sup35 oligopeptide repeats, and by extension PrP repeats, promote prion propagation *in vivo* is poorly understood. To date, three models, which are not mutually exclusive, have been proposed. First, repeats may promote the conversion of soluble protein to the amyloid state, an idea that is consistent with the increased accumulation of soluble Sup35 upon repeat deletion [36]. Second, repeats may stabilize Sup35-Sup35 interactions outside of the conversion interaction, which dictates the amyloid core [17]. Consistent with this model, repeat sequences have been shown to mediate Sup35-Sup35 interactions and to be partially protected from solvent exchange in amyloid fibers *in vitro* [37–39]. Finally, repeats may facilitate chaperone interactions by serving as direct binding sites or by altering the conformation of the amyloid to allow chaperone access [16], a prediction that is supported by the observation that changes in aggregate size, an attribute linked to chaperone processing [40], inversely correlate with repeat number [36].

Distinguishing among these models has been challenging. Previous studies have relied on steady-state observations, but prion propagation in yeast is a multistep process that requires prion protein synthesis, its conversion to the amyloid state via interaction with existing amyloid, the fragmentation of growing amyloid aggregates by molecular chaperones, and the transmission of these complexes to daughter cells [2].

Here, we address the specific mechanistic contributions of the Sup35 oligopeptide repeats to prion propagation *in vivo*. We find that this region is composed of two functional elements, the oligopeptide repeats themselves and a downstream region, which we refer to as the repeat proximal region (RPR). Without significantly altering the kinetic stability of the amyloid state, the presence of the oligopeptide repeats and the RPR primarily promote chaperone-mediated fragmentation of Sup35 aggregates. But, these elements likely act through distinct mechanisms, with the RPR promoting chaperone binding, and the oligopeptide repeats promoting chaperone processing.

## Results

### Oligopeptide repeats act synergistically with a newly identified sequence element to enhance the severity of the [*PSI*^*+*^] phenotype

Wildtype Sup35 contains five and a half oligopeptide repeats [41,42]. But, previous analyses of their contribution to prion propagation *in vivo* used deletion constructs that also removed the RPR, a downstream asparagine-rich region (amino acids 98–111, RGNYKNFNYNNNLQ). Thus, the potential contributions of each element to prion propagation have not been disentangled.

**Fig 1:**
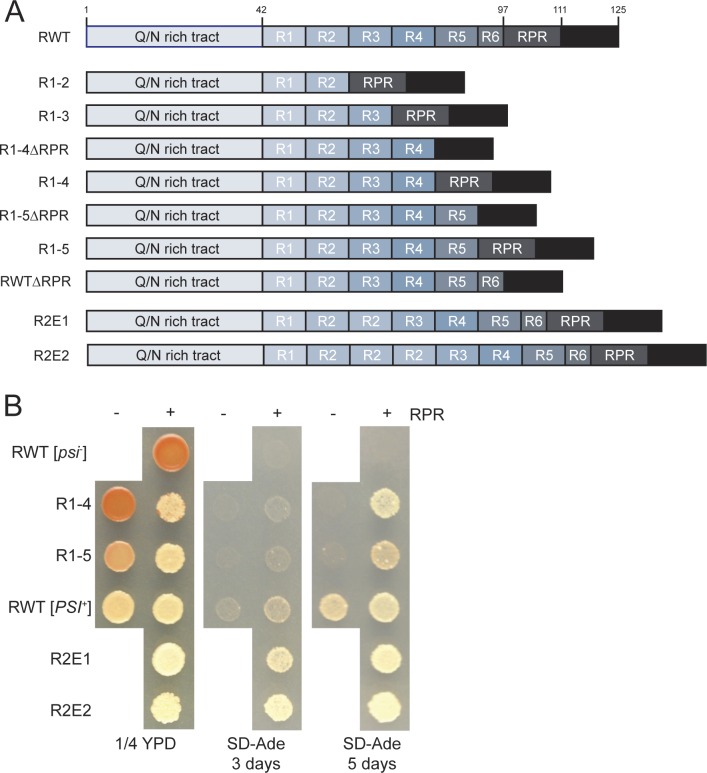
[*PSI*^*+*^] maintenance depends on two sequence elements in the oligopeptide repeat region. **A.** Schematic diagram of repeat variant constructs. See text for abbreviations. **B.** Sequence variant strains (R1-4 (SY2057), R1-5 (SY2022), R2E1 (SY2247), R2E2 (SY2300), R1-4ΔRPR (SY1629), R1-5ΔRPR (SY1633), and ΔRPR (SY2023)) were spotted onto rich medium (1/4 YPD), or medium lacking adenine (SD-Ade) to analyze the [*PSI*^*+*^] phenotype. Wildtype [*psi*^*-*^] and [*PSI*^*+*^] strains (SLL2119 and SLL2606, respectively) are shown as controls.

To separately assess the contribution of the Sup35 oligopeptide repeat region to [*PSI*^*+*^] propagation *in vivo*, we constructed a series of yeast strains expressing repeat sequence variants (RVs), composed of full-length Sup35 containing a different number of repeats (Fig 1A). This collection includes repeat deletion strains (R1-X), which contain the N terminal X repeats, from 2 to 5, and repeat expansion strains (R2E1 and R2E2), which contain one or two extra copies of the second repeat for a total of 6.5 or 7.5 repeats, respectively. The RVs replaced the wildtype copy of Sup35 at the endogenous locus and were expressed from the Sup35 promoter to wildtype levels (S1A Fig). However, using this same configuration, the repeat expansion variants (R2E1 and R2E2) were expressed at a much lower level. Thus, we integrated a second copy of the repeat expansion proteins expressed from the *SUP35* (R2E1) or *MFA1* (R2E2) promoters at another locus in these strains to raise their expression to wildtype levels (S1A Fig). Because the extent to which this region of the protein is buried in the fiber interface varies based on the conformation of the protein in the amyloid [37], we have restricted our analysis to the [*PSI*^*+*^]^Strong^ variant of Sup35.

To assess the prion phenotypes of our RV strains, we took advantage of the premature termination codon (PTC) in the *ADE1* gene in our yeast strain background (the *ade1-14* allele), which provides a sensitive reporter of Sup35 translation release factor activity *in vivo* [43]. Soluble non-prion Sup35 promotes efficient translation termination at the PTC in [*psi*^-^] strains, while aggregated Sup35 in [*PSI*^*+*^] strains is functionally compromised, allowing read through of the PTC. These differences in Ade1 expression cause [*psi*^-^] strains to form red colonies on rich medium and block their growth on minimal medium lacking adenine, while [*PSI*^*+*^] strains form white colonies on rich medium and can grow on minimal medium lacking adenine [44]. Changes in [*PSI*^*+*^] propagation efficiency, which lead to the accumulation of increased soluble and decreased aggregated Sup35, result in the formation of pink colonies on rich medium and partial growth on medium lacking adenine [45].

The phenotypes of strains containing four, five, or the wildtype number of repeats were nearly indistinguishable on rich medium (Fig 1B), but the strain expressing R1-4 lost the [*PSI*^*+*^] prion at a frequency of ~3%, while [*PSI*^*+*^] was fully stable in the other strains. When the number of repeats was reduced, the colonies grew slightly worse on medium lacking adenine compared to the wildtype strain (Fig 1B). In contrast, when the number of repeats was expanded, the colonies appeared more white on rich medium and grew slightly better on medium lacking adenine, indicating more efficient stop codon read-through (Fig 1B). Repeat deletion strains containing only 2 or 3 repeats (R1-2 and R1-3, respectively) were unable to support [*PSI*^*+*^] (S1B and S1C Fig), indicating that at least four repeats are required for prion maintenance.

Our observations are in conflict with previous studies, in which [*PSI*^*+*^] maintenance was reported to require the presence of at least five repeats [16,17], suggesting the RPR may play a role in prion propagation *in vivo*. Indeed, deletion of the RPR in our wildtype construct modestly reduced the strength of the [*PSI*^*+*^] phenotype, producing colonies that were more pink on rich medium and that grew at a reduced rate on medium lacking adenine (Fig 1B). This same trend was also apparent when the RPR was removed from the repeat deletion strains (Fig 1B). Notably, while a construct containing only 4 repeats was unable to maintain [*PSI*^*+*^] in the absence of the RPR, as previously reported [16,17], it regained the ability to do so in the presence of the RPR (Fig 1B and S1D Fig). Thus, removal of the RPR enhances the prion propagation defect caused by deletion of Sup35 repeats.

### The repeats and RPR do not significantly alter conversion efficiency

The defect in prion propagation induced by deletion of the repeats or the RPR suggests a change in some aspect of prion aggregate dynamics *in vivo*: conversion of soluble Sup35 to the amyloid form and/or amyloid fragmentation by the molecular chaperones Hsp104, Hsp70 (Ssa1), and Hsp40 (Sis1) [2]. To identify the defects specific to each sequence variant, we first characterized a subset of strains, which represented each type of alteration: R1-5 (repeat deletion), R2E1 (repeat expansion), and ΔRPR (RPR deletion). For the repeat deletions and expansions, we first chose the most conservative changes to minimize toxicity, as doubling times increased with more severe changes in the number of these sequence elements (Table 1).

**Table 1:** Doubling times (in minutes) of repeat and RPR variant strains grown in rich medium.

	[*PSI*^*+*^]	[*PSI*^*+*^] + Sup35C
**RWT**	97.7 ± 4.1	96.4 ± 0.8
**ΔRPR**	99.5 ± 0.6	n.a.
**R1-4**	126.5 ± 1.5	97.9 ± 2.1
**R1-5**	99.1 ± 3.2	n.a.
**R2E1**	112.3 ± 2.6	n.a.
**R2E2**	166.0 ± 1.4	n.a.

**Fig 2:**
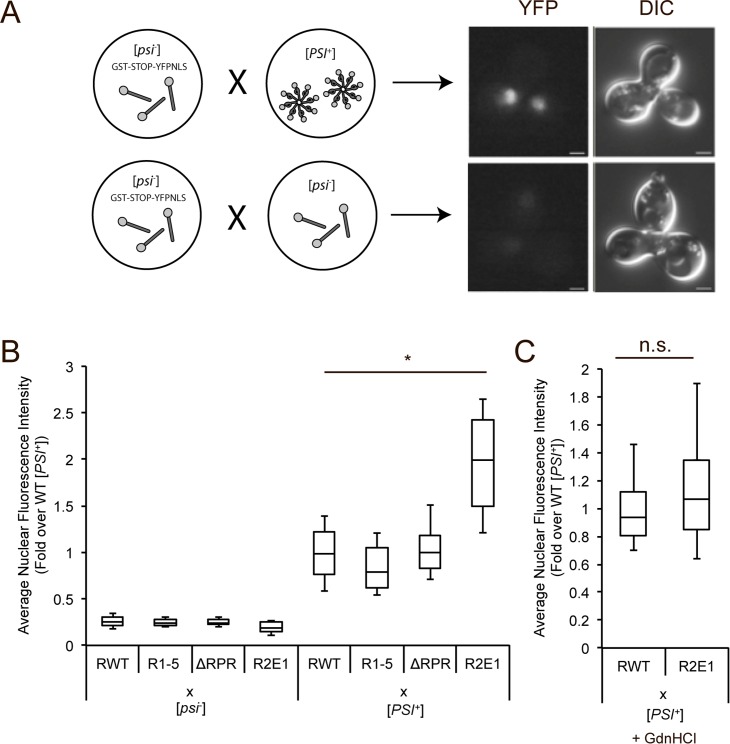
Neither Sup35 repeats nor the RPR impact the efficiency of prion conversion. **A.** Schematic representation of conversion mating assay. Wildtype [*psi*^*-*^] strain containing the read-through reporter GST(UGA)YFP-NLS (SY2393) was mated to wildtype [*PSI*^*+*^] (SLL2606, top) or [*psi*^*-*^] (SY2119, bottom). Ball and sticks represent soluble non-prion Sup35; pinwheels represent prion Sup35 amyloid. **B.** Wildtype (SY2393), R1-5 (SY2461), ΔRPR (SY2463), and R2E1 (SY2465) [*psi*^*-*^] strains containing the read-though reporter GST(UGA)YFP-NLS were mated to wildtype [*psi*^*-*^] or [*PSI*^*+*^] (SLL2119 and SLL2606, respectively). Nuclear fluorescence intensity of zygotes was quantified. Horizontal lines on boxes indicate 25^th^, 50^th^, and 75^th^ percentiles. Whiskers indicate 10^th^ and 90^th^ percentiles. n≥21, *p = 10^−23^,student’s t-test. **C.** Wildtype (SY2393) and R2E1 (SY2465) strains containing the read-through reporter GST(UGA)YFP-NLS were mated to wildtype [*PSI*^*+*^] (SLL2606) in the presence of guanidine HCl (GdnHCl). Nuclear fluorescence intensity of zygotes was determined. Box plots are as described in panel B. n≥38, n.s. not significant.

Using these strains, we first directly monitored the conversion of soluble Sup35 to the amyloid state using a fluorescent read-through assay, which reports on the defect in Sup35 translation release activity upon aggregation of the protein [46,47]. In this assay, [*psi*^*-*^] strains, expressing one of the sequence variants and the read-through reporter GST(UGA)YFP-NLS, were mated to a wildtype [*PSI*^*+*^] strain. While YFP is not initially expressed in the reporter strain due to the absence of [*PSI*^*+*^], the conversion of the soluble Sup35 protein in the [*psi*^-^] strain to the amyloid state upon mating to the [*PSI*^*+*^] partner leads to the accumulation of nuclear fluorescence in the zygote (Fig 2A). As expected, in zygotes formed by crossing the R1-5, ΔRPR, R2E1 or wildtype [*psi*^-^] strains to a wildtype [*psi*^-^] strain, nuclear fluorescence was low and nearly identical (Fig 2B, lanes 1–4), indicating that the Sup35 sequence variants in these crosses remain soluble and functional. In contrast, when the R1-5, ΔRPR or wildtype [*psi*^-^] strains were crossed to a wildtype [*PSI*^*+*^] strain, nuclear fluorescence was higher than seen in the cross to a wildtype [*psi*^-^] strain but was still similar among the three crosses (Fig 2B, lanes 5–7), indicating that the Sup35 sequence variants containing a repeat or RPR deletion are not significantly impaired in their ability to convert to the amyloid state. However, the R2E1 [*psi*^-^] X wildtype [*PSI*^*+*^] cross produced nuclear fluorescence intensity that was significantly increased relative to the wildtype zygote (Fig 2B, compare lanes 5 and 8), suggesting, at face value, that the repeat expansion may more readily convert to the amyloid form, an observation consistent with the idea that the repeats promote conversion [36].

Because differences in amyloid fragmentation by molecular chaperones affect the accumulation of templates and thereby the conversion efficiency of soluble protein [48], we hypothesized that the difference in nuclear fluorescence intensity observed in the R2E1 heterozygous zygote was a downstream effect of an increase in fragmentation efficiency (i.e. more templates support more conversion). To test this hypothesis, we repeated the wildtype and R2E1 [*psi*^-^] crosses to a wildtype [*PSI*^*+*^] strain in the presence of guanidine HCl (GdnHCl), which inhibits Hsp104 activity, blocks fragmentation, and reduces the number of amyloid templates [49,50]. In the presence of GdnHCl, the nuclear fluorescence intensity was similar in zygotes produced by crossing either a wildtype or R2E1 [*psi*^-^] strain with a wildtype [*PSI*^*+*^] strain (Fig 2C). Because the increased nuclear fluorescence intensity of the R2E1 strain was abolished upon Hsp104 inhibition, we conclude that the R2E1 protein converts to the aggregated state with similar efficiency to wildtype Sup35.

### The repeats and RPR act at the fragmentation step of prion propagation

**Fig 3:**
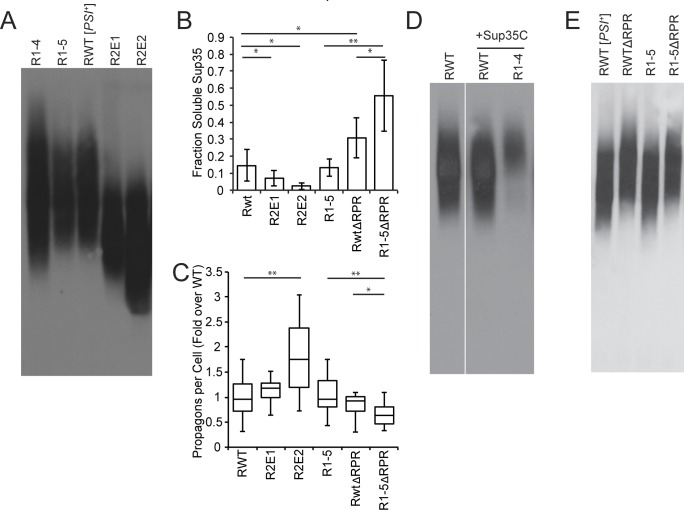
Sup35 repeats and RPR promote amyloid fragmentation. **A.** SDD-AGE was performed on R1-4 (SY2057), R1-5 (SY2022), wildtype [*PSI*^*+*^] (SLL2606), R2E1 (SY2247), R2E2 (SY2300) lysates, followed by immunoblotting for Sup35. **B.** Cell lysates from wildtype [*PSI*^*+*^] (SLL2606), R2E1 (SY2247), R2E2 (SY2300), R1-5 (SY2022), ΔRPR (SY2023), and R1-5ΔRPR (SY1633) were incubated at 53°C and 100°C in the presence of SDS before SDS-PAGE and immunoblotting for Sup35. The amount of soluble Sup35 was determined as the percentage of signal at 53°C relative to 100°C. Bars represent means, error bars represent standard deviations. n≥5, *p<0.05, **p<0.01, student’s t-test. **C.** The number of propagons in wildtype [*PSI*^*+*^] (SLL2606), R2E1 (SY2247), R2E2 (SY2300), R1-5 (SY2022), ΔRPR (SY2023), and R1-5ΔRPR (SY1633) strains was determined by an *in vivo* colony based dilution assay. Horizontal lines on boxes represent the 25^th^, 50^th^, and 75^th^ percentiles. Whiskers represent maximum and minimums. n≥13, *p<0.05, **p<0.01, student’s t-test. **D.** SDD-AGE was performed on wildtype [*PSI*^*+*^] (SLL2606), wildtype [*PSI*^*+*^] expressing an extra copy of Sup35C (SY2466) and R1-4 expressing an extra copy of Sup35C (SY2467) lysates, followed by immunoblotting for Sup35. Panels represent non-consecutive lanes run on the same gel. **E.** SDD-AGE was performed on wildtype [*PSI*^*+*^] (SLL2606), ΔRPR (SY2023), R1-5 (SY2022), and R1-5ΔRPR (SY1633) lysates, followed by immunoblotting for Sup35.

While the striking and Hsp104-dependent increase in nuclear fluorescence observed in the R2E1 [*psi*^-^] X wildtype [*PSI*^*+*^] cross did not obviously correspond to conversion propensity, it did suggest that the R2E1 amyloid was fragmented at an increased efficiency relative to the wildtype and RV deletion amyloids (Fig 2B and 2C). If true, we would expect the R2E1 protein to accumulate more and smaller aggregates at steady-state, and this increase in template number would in turn decrease the level of soluble Sup35 at steady-state. Indeed, by semi-denaturing detergent agarose gel electrophoresis (SDD-AGE) [51], the R2E1 protein accumulated in smaller SDS-resistant aggregates than wildtype Sup35 (Fig 3A), and this change correlated with a decrease in SDS-sensitive (i.e. soluble) R2E1 protein in comparison with wildtype Sup35 (Fig 3B). This decrease in soluble protein in the R2E1 strain should also induce a more severe translation termination defect, a prediction consistent with its colony-based phenotypes: whiter on rich medium and more robust growth on medium lacking adenine (Fig 1B).

Surprisingly, we could not detect an increase in heritable R2E1 aggregates (known as propagons) using a genetic assay based on the transfer of existing aggregates to daughter cells in the absence of fragmentation [52] (Fig 3C). We reasoned that this observation could be explained by the fact that the shift in the size distribution of R2E1 aggregates was slight relative to wildtype Sup35 (Fig 3A) and therefore beyond the sensitivity of this genetic-based assay. But, similar analyses of the R2E2 strain demonstrated a more severe shift in size to smaller SDS-resistant aggregates by SDD-AGE (Fig 3A), a further decrease in SDS-sensitive protein (Fig 3B) and a corresponding increase in propagons (Fig 3C). Thus, expansion of the number of repeats in the Sup35 protein likely promotes amyloid fragmentation.

Consistent with this idea, deletion of repeats 4 and 5 in the context of R2E2, which returns the total number of repeats to 5.5, shifts the steady-state size of Sup35 aggregates to a near wildtype distribution (S1A and S1E Fig), suggesting that repeat number rather than identity is the dominant force in amyloid fragmentation. If true, deletion of repeats below the wildtype number should further decrease amyloid fragmentation. To assess this prediction, we performed similar analyses on the R1-5 strain, which removed one repeat and decreased the efficiency of [*PSI*^*+*^] propagation *in vivo* (Fig 1). Surprisingly, the steady-state size distribution of aggregates (Fig 3A), the level of soluble protein (Fig 3B), and the number of propagons (Fig 3C) were indistinguishable between the R1-5 and wildtype strains.

Given our observations of progressive effects in the repeat expansion strains, we next analyzed the size distribution of aggregates in the R1-4 strain, which removed one additional repeat from R1-5 (Fig 1A). Rather than observing a simple shift in aggregates to a larger size distribution, we observed a broadening of entire size distribution when R1-4 aggregates were analyzed by SDD-AGE (Fig 3A). In contrast to the wildtype strain, [*PSI*^*+*^] propagated by the R1-4 protein induces a strong extension of the strain doubling time (Table 1), suggesting it is a toxic state that could indirectly alter amyloid dynamics through changes in protein homeostasis (proteostasis) [53]. To circumvent this possibility, we expressed, in the R1-4 strain, the domain of Sup35 that functions in translation termination (amino acids 254–685; Sup35-C), which had been previously demonstrated to relieve the toxicity induced by overexpression of the Sup35 prion domain (amino acids 1–253; Sup35-NM) [54]. Expression of Sup35-C eliminated the toxicity of [*PSI*^*+*^] propagated by the R1-4 protein (Table 1), and the R1-4 protein accumulated in larger aggregates than those of the wildtype protein in a [*PSI*^*+*^] strain that also expressed Sup35-C (Fig 3D). This observation is consistent with the idea that deletion of repeats decreases the efficiency of fragmentation *in vivo*.

We next assessed the effects of deletion of the RPR on fragmentation, given its synergistic effects on [*PSI*^*+*^] propagation with repeat deletions (Fig 1B). In the context of a wildtype number of repeats, deletion of the RPR slightly shifted the aggregate size distribution to larger complexes (Fig 3E). This change in size did not significantly alter the number of propagons (Fig 3C), perhaps again reflecting the limited sensitivity of the assay, but it did increase the accumulation of soluble ΔRPR protein in comparison with wildtype (Fig 3B). These observations, in combination with the direct demonstration that the ΔRPR protein converts to the aggregated state with an efficiency that is not significantly different from wildtype (Fig 2B), suggest that RPR deletion reduces the number of amyloid templates and therefore the efficiency of fragmentation.

Because deletion of the RPR exacerbated the effects of repeat deletion on [*PSI*^*+*^] propagation (Fig 1B), we reasoned that the ΔRPR protein might sensitize our assays to more directly reveal a fragmentation defect for R1-5 amyloid. Deletion of the RPR in the R1-5 protein did not shift the distribution of aggregates to larger sizes beyond its effect in the wildtype protein (Fig 3E). But, it did increase the soluble protein (Fig 3B, compare lanes 5 and 6) and decrease the number of propagons (Fig 3C, compare lanes 5 and 6) in comparison with deletion of the RPR alone. Thus, removing a single half repeat is sufficient to reduce amyloid fragmentation efficiency in the ΔRPR strain.

To directly assess the rate of amyloid fragmentation *in vivo*, we turned to a propagon amplification assay. In this assay, strains are grown in rich medium containing GdnHCl, which reversibly inhibits Hsp104 [50], to reduce Sup35 aggregate number to the point just before [*psi*^-^] cells appear in the population. Strains are then allowed to recover in rich medium in the absence of GdnHCl, where the re-amplification of existing aggregates can occur upon Hsp104 reactivation [55]. The rate of this re-amplification had been previously linked to the product of the conversion and fragmentation rates [55], but since our sequence variants have similar conversion efficiencies (Fig 2B and 2C), this amplification rate provides a proxy for relative fragmentation rate.

**Fig 4:**
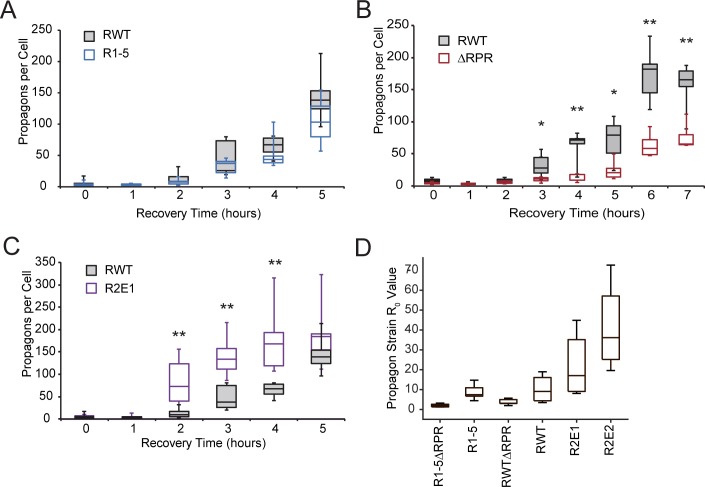
Sequence variant strains differ in their ability to amplify Sup35 amyloid. **A.** Wildtype [*PSI*^*+*^] (SLL2606) and R1-5 (SY2022) strains were grown in YPAD + 3mM GdnHCl until nearly cured, followed by growth in YPAD for the indicated times. At each time point, the number of propagons per cell was determined. Horizontal lines on boxes indicate 25^th^, 50^th^, and 75^th^ percentiles, and whiskers indicate maximum and minimum. n≥4. **B.** Wildtype [*PSI*^*+*^] (SLL2606) and ΔRPR (SY2023) strains were treated as in A. n≥4, *p<0.05, **p<0.01, student’s t-test. **C.** Wildtype [*PSI*^*+*^] (SLL2606) and R2E1 (SY2247) strains treated as in A. n≥4, **p<0.01, students t-test. **D.** Mathematical modeling was used to calculate the *R*_*0*_ value for the indicated strains using the steady-state soluble Sup35 levels (Fig 3B). Box plots are as described in panel A. Statistical analysis is described in the Supplementary Materials.

Following release from GdnHCl, individual cells were isolated at the indicated time points, and the number of propagons per cell was determined. Importantly, the number of propagons in each strain was similar prior to GdnHCl treatment (Fig 3C), and GdnHCl treatment similarly reduced propagon number in all strains (Fig 4A–4C, 0 time point). The R1-5 protein subtly, but reproducibly, recovers its propagon levels more slowly than wildtype (Fig 4A). For the ΔRPR and R2E1 strains, propagon amplification is strongly and significantly different than wildtype, with the ΔRPR strain having a reduced and the R2E1 strain supporting an enhanced rate of recovery relative to the wildtype strain (Fig 4B and 4C). These activities are consistent with our observations at the colony (Fig 1B), protein (Figs 2B, 3A and 3B), and propagon (Fig 3C) levels.

While it is formally possible that these mutants differ from wildtype in their conversion efficiencies to a degree that is undetectable by our fluorescence-based assay (Fig 2), the predicted changes in their efficiencies based on the shifts in steady-state size of Sup35 aggregates are opposite of what would be predicted based on their rates of aggregate amplification. For example, an increase in aggregate size (i.e. ΔRPR Fig 3E) should correspond to an increase in conversion efficiency [40] and in turn an increase in amplification rate [55], predictions that are not born out by our observations (Fig 4). Thus the combination of observations can be most parsimoniously reconciled with a model in which the dominant contributions of both the repeats and the RPR are to promote amyloid fragmentation *in vivo*.

To assess the impact of these sequence elements on fragmentation rates using another method, we developed a mathematical model of aggregate amplification, capable of determining the basic reproductive number of aggregates (*R*_*0*_), which is the number of additional aggregates produced by each aggregate in its lifetime, using a different experimental input parameter: the steady-state level of soluble Sup35 (S1 Table). *R*_*0*_ is proportional to the fragmentation rate (γ), when the rates of synthesis (α) and dilution (μ) and minimum aggregate size (*n*_*0*_) are held constant (see Supplementary Materials). The repeat expansion (R2E2) had a higher *R*_*0*_ and the RPR deletion had a lower *R*_*0*_ than wildtype Sup35 (Fig 4D and S2 Table), consistent with a direct effect on rate of fragmentation or an inverse effect on the minimum aggregate size, respectively. While we cannot rule out a change in *n*_*0*_ for these mutants relative to wildtype, we also cannot explain the wholesale shifts in the steady-state size distributions of the Sup35 aggregates for these mutants (Fig 3A) without a change in fragmentation [40]. As was the case for the propagon amplification assay (Fig 4A), the wildtype and R1-5 strains could not be distinguished by this method (Fig 4D and S2 Table). However, the *R*_*0*_ value for R1-5ΔRPR was smaller than that of ΔRPR alone (Fig 4D and S2 Table), suggesting, that deletion of the half repeat also reduced the rate of amyloid fragmentation *in vivo*.

**Fig 5:**
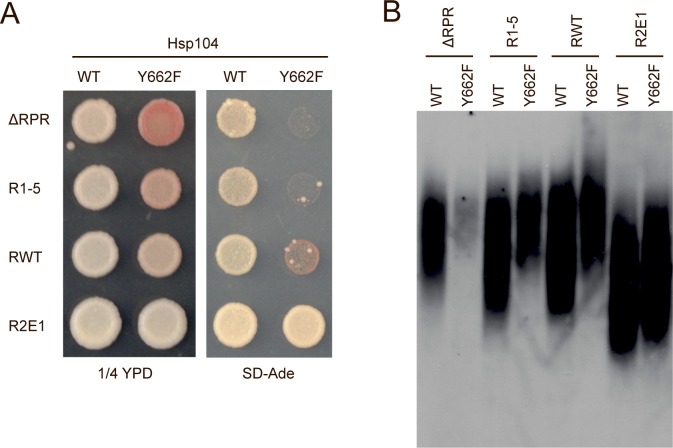
Sup35 sequence variant strains show synergistic effects with an Hsp104 pore mutant. **A.** Sequence variant strains expressing Hsp104WT (ΔRPR (SY2023), R1-5 (SY2022), RWT (SLL2606), R2E1 (SY2247)) or Hsp104Y662F (ΔRPR (SY3004), R1-5 (SY3002), RWT (SY3001), and R2E1 (SY3005)) were spotted on rich medium (1/4 YPD) and medium lacking adenine (SD-Ade) to analyze the [*PSI*^*+*^] phenotype. **B.** SDD-AGE was performed on lysates from the strains described in A, followed by immunoblotting for Sup35.

Fragmentation is catalyzed by the molecular chaperone Hsp104 [48,50,56–58], and Sup35 sequence elements impacting fragmentation efficiency would therefore act through this catalyst. As such, their deletion or expansion would be predicted to have distinct genetic interactions with an Hsp104 mutant (Y662F) having a reduced efficiency of substrate processing [59]. Specifically, Sup35 sequence changes that inhibit fragmentation should enhance the Hsp104^Y662F^ defect, while those that promote fragmentation should suppress the Hsp104^Y662F^ defect. As previously reported, Hsp104^Y662F^ reduces the efficiency of [*PSI*^*+*^] propagation by wildtype Sup35 [60], leading to the formation of pinker colonies on rich medium, a reduced growth rate on medium lacking adenine (Fig 5A), and a shift in aggregate distribution to larger size by SDD-AGE (Fig 5B). This Hsp104^Y662F^ defect was suppressed by the R2E1 protein, which has a higher fragmentation rate *in vivo* (Fig 4C and S2 Table), both at the colony level (Fig 5A) and by aggregate size (Fig 5B). In contrast, the Hsp104^Y662F^ defect was enhanced by deletion of prion domain sequence elements. Both the R1-5 and ΔRPR strains form colonies that are more pink on rich medium and that are unable to grow on medium lacking adenine in the presence of Hsp104^Y662F^ (Fig 5A), and aggregates of these proteins are shifted to larger size distributions in the presence of Hsp104^Y662F^ (Fig 5B). The latter effect is especially dramatic for the RPR deletion, where Hsp104^Y662F^ nearly eliminates Sup35 amyloid (Fig 5B). Taken together, our data indicate that the repeat and RPR regions function in the Sup35 protein to promote amyloid fragmentation.

### Sup35 sequence variants do not interfere with [*PSI*^*+*^] propagation through significant changes in the expression of fragmentation chaperones or the physical properties of aggregates

**Fig 6:**
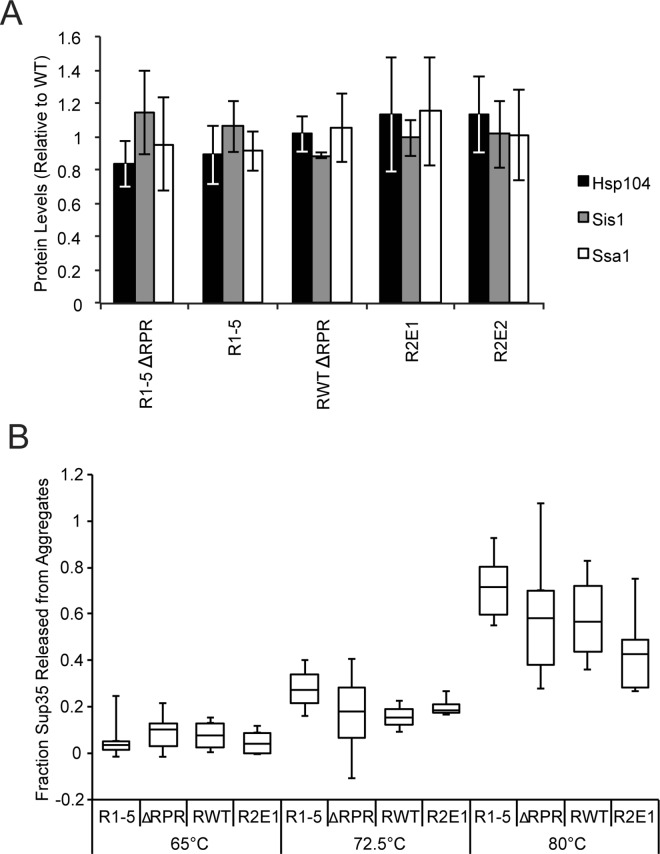
Sup35 repeat number and the RPR do not affect steady-state chaperone levels or aggregate kinetic stability. **A.** R1-5ΔRPR (SY1633), R1-5 (SY2022), ΔRPR (SY2023), R2E1 (SY2247), R2E2 (SY2300) were analyzed by SDS-PAGE and quantitative immunoblotting for Hsp104 (black), Ssa1 (gray), and Sis1(white). Bars represent means; error bars represent standard deviations. n≥3. **B.** Lysates from R1-5 (SY2022), ΔRPR (SY2023), wildtype [*PSI*^*+*^] (SLL2606), and R2E1 (SY2247) strains were incubated in 2% SDS at the indicated temperatures before SDS-PAGE. The percent of Sup35 released from aggregates at each temperature was determined relative to a sample incubated at 53°C. Horizontal lines on boxes represent 25^th^, 50^th^, and 75^th^ percentiles. Whiskers represent maximums and minimums. n≥3.

Having identified fragmentation as the dominant step of prion propagation that is impacted by the repeats and the RPR, we next sought to determine the mechanism by which these sequence elements act. Because expression of the more extensive repeat expansion and deletion constructs was toxic (Table 1), we first considered the possibility that the sequence variants might induce a stress response, resulting in altered chaperone levels, substrate load and amyloid fragmentation [61–63]. However, the expression levels of Hsp104 and its co-chaperones Ssa1 (Hsp70) and Sis1 (Hsp40) in our RV and ΔRPR strains were similar to wildtype (Fig 6A). Therefore, changes in the levels of the chaperones that catalyze amyloid fragmentation are unlikely to explain the differences in fragmentation efficiency in these strains.

We next considered the possibility that the repeats and the RPR may promote fragmentation by altering the kinetic stability of Sup35 amyloid, as has been observed for the Sup35 G58D mutant [46,64]. Indeed, combined repeat and RPR truncations assemble into amyloid with higher kinetic stability when overexpressed [65]. However, the [*PSI*^*+*^] prion variant used in that study was distinct from the [*PSI*^*+*^] strong strain used in these studies [36,65], and Sup35 sequence variants are known to confer conformation-specific effects on amyloid [64,66]. To determine the kinetic stability of Sup35 amyloid in our strains, we assessed their solubility in 2% SDS over a range of temperatures by their ability to enter an SDS-polyacrylamide gel [67]. As the temperature was increased from 65°C to 80°C, the amount of SDS-soluble Sup35 increased, as expected [67,68], due to amyloid disassembly (Fig 6B). But across this temperature range, the amount of SDS-soluble R1-5, ΔRPR and R2E1 protein was similar to that of wildtype Sup35 (Fig 6B). Thus, the changes in protein sequence did not significantly alter the kinetic stability of amyloid or, by extension, their conformation.

### The repeat region and the RPR alter the interaction between Sup35 and molecular chaperones

Because the relative chaperone levels and kinetic stability of Sup35 amyloid are not significantly altered in the repeat or RPR sequence variant strains, we next considered the possibility that these sequence elements might promote fragmentation by affecting the efficiency with which Sup35 amyloid is recognized or processed by molecular chaperones. We expressed HA-tagged Sup35 prion domain (NM-HA) from the Sup35 promoter from a single-copy integrated construct, using repeat and RPR variants of this fragment to match the full-length protein expressed in each strain. Importantly, the NM-HA protein was expressed at similar levels in all strains (S1A Fig). For the R1-5 and the R2E1 strains, the size of SDS-resistant aggregates was similar in the presence (S2B Fig) and absence (Fig 3A) of NM-HA. However, for the ΔRPR strain, expression of NM-HA eliminated the shift in SDS-resistant aggregate size (S2B Fig) that we observed in its absence (Fig 3E), suggesting that expressing the prion domain as a separate fragment perturbs amyloid dynamics *in vivo*. However, expression of the ΔRPR variant as an HA-tagged full-length Sup35 (NM-HA-C) protein (S2C Fig) recapitulated the shift in the size distribution of SDS-resistant aggregates to larger complexes by SDD-AGE (Fig 3E and S2D Fig).

**Fig 7:**
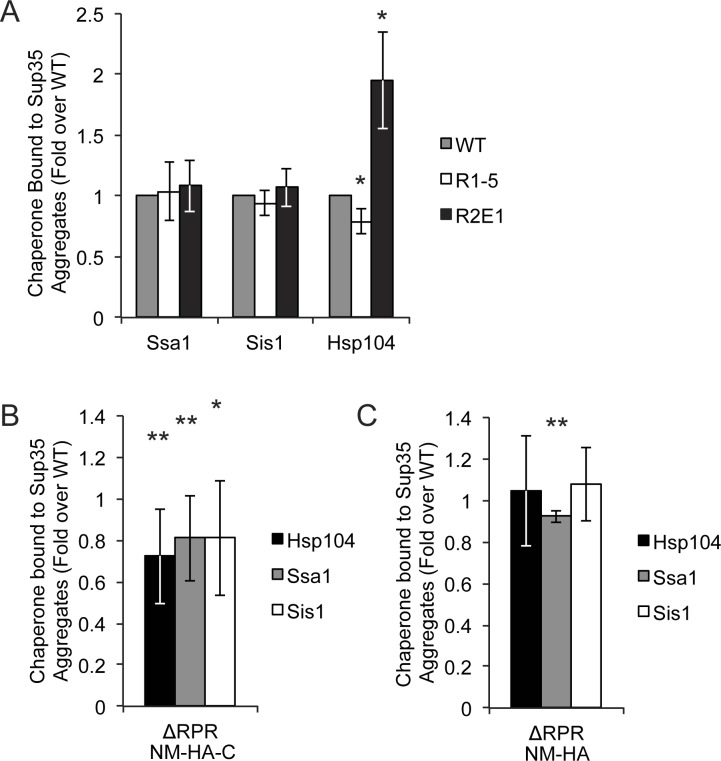
Chaperone binding to Sup35 amyloid differs in sequence variant strains. **A.** NM-HA and co-aggregating full-length (untagged) Sup35 in wildtype (SY3007), R1-5 (SY3008), and R2E1 (SY3010), strains were immunocaptured using anti-HA magnetic beads and separated by SDS-PAGE, and the amount of bound chaperone proteins was quantified by immunoblotting and corrected for the amount of Sup35 present in aggregates in each strain (see methods). Bars represent means; error bars represent standard deviations, n≥4, *p<0.05, student’s t-test. **B.** NM-HA-C and co-aggregating full-length Sup35 in wildtype (SY3159) and ΔRPR (SY3164) strains were immunocaptured with anti-HA magnetic beads and separated by SDS-PAGE, and the amount of bound chaperones was quantified by immunoblotting and corrected for the amount of Sup35 present in aggregates. Bars represent means; error bars represent standard deviations. n≥8, *p<0.05, **p<0.01, student’s t-test. **C.** NM-HA and co-aggregating full-length (untagged) Sup35 in wildtype (SY3007) and ΔRPR (SY3009) strains were immunocaptured with anti-HA magnetic beads and separated by SDS-PAGE, and the amount of bound chaperones was quantified by western blot and corrected for the amount of Sup35 present in aggregates. Bars represent means; error bars represent standard deviations. n = 4, **p<0.01, student’s t-test.

Using quantitative co-immunocapture of the HA-tagged Sup35 sequence variants, similar levels of Ssa1 and Sis1 were bound to the R1-5, R2E1, and wildtype proteins (Fig 7A and S3A Fig). However, Hsp104 binding was altered by changes in repeat number. When repeats were deleted (R1-5), slightly but significantly less Hsp104 was bound, but when repeats were expanded (R2E1) the amount of bound Hsp104 increased (Fig 7A and S3A Fig). For the ΔRPR protein, binding to all three chaperones, Hsp104, Ssa1 and Sis1, was reduced by ~20% in comparison with wildtype Sup35 using the full-length HA-tagged protein (Fig 7B and S3B Fig). Thus, chaperone binding correlates directly with fragmentation efficiency for the Sup35 sequence variants.

### The RPR and repeat region make distinct contributions to substrate outcomes *in vivo*

While our binding studies revealed that changes in both the repeats and the RPR alter the interaction of Sup35 with chaperones, their targets were distinct, with the number of repeats directly correlating only with Hsp104 binding and deletion of the RPR lowering the binding of all three chaperones (Fig 7A and 7B). Sup35 amyloid fragmentation is believed to be initiated by the binding of these complexes to Sis1 and Ssa1 and their subsequent transfer to Hsp104 [69–71]. Thus, one interpretation of these observations is that the RPR promotes initial chaperone binding, but that once the amyloid is transferred to Hsp104, the repeats impact the efficiency with which Hsp104 processes this substrate, with lower processing corresponding to lower binding at steady-state.

If the primary function of the RPR is to promote initial chaperone binding, why would this contribution become unnecessary in the context of the NM protein? In comparison with full-length Sup35, the truncated prion domain completely lacks a stable, folded domain. As such, it could be recognized more easily by the chaperone machinery, making the contribution of the RPR less important. Indeed, more Ssa1, Sis1 and Hsp104 bound to ΔRPR NM-HA (Fig 7C and S3A Fig) than to ΔRPR NM-HA-C (Fig 7B and S3C Fig). Thus, increasing chaperone binding by removing the functional C-terminal domain can compensate for the ΔRPR prion propagation defect.

In light of this observation, we reasoned that we could distinguish between binding and processing events by inserting the Sup35 prion domain fragments into another Hsp104 substrate. If the Sup35 fragment impacted substrate processing efficiency, it should similarly affect Hsp104 action on this substrate. However, if the Sup35 fragment promoted chaperone binding, it may not affect Hsp104 action because the *bona fide* substrate already effectively recruits chaperones in the absence of this fusion, in the same way that deletion of the RPR had no effect in the context of the isolated prion domain (Fig 7C and S2B and S3A Figs). To test this idea, we modified an existing microscopy-based assay, in which a folding sensor composed of firefly luciferase fused to GFP is expressed in yeast cells, induced to misfold and aggregate by heat shock, and allowed to disaggregate and refold during recovery at normal temperature in an Hsp104-dependent manner [72,73].

**Fig 8:**
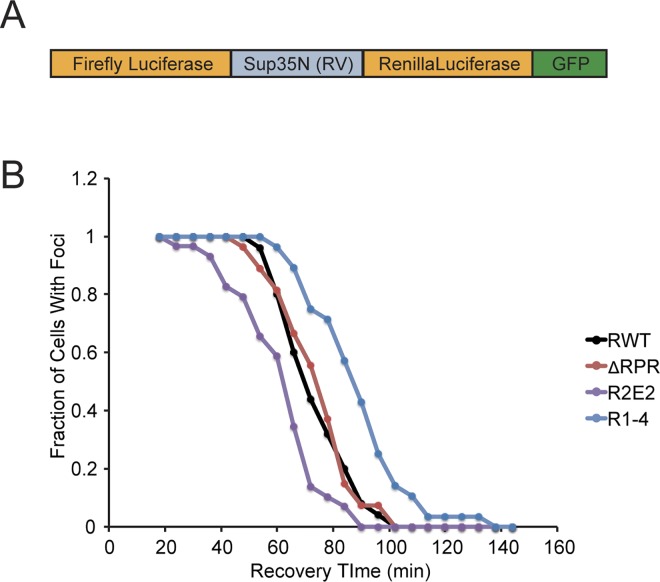
Sup35 repeats but not RPR exert transferrable effects on another Hsp104 substrate. **A.** Schematic diagram of reporter construct. **B.** The indicated strains expressing luciferase reporter (RWT (SY2603), R1-4 (SY2666), R2E2 (SY2640), and ΔRPR (SY2637)) constructs were incubated at 37°C for 30 minutes, followed by 40°C for 35 minutes, with cycloheximide added for the last 10 minutes, in a microfluidics chamber. Cells were then allowed to recover at 30°C in the presence of cycloheximide, and images were taken at the indicated time points. At each time point, the percentage of cells containing GFP foci was determined.

Other members of AAA+ ATPase chaperone family, to which Hsp104 belongs, have been shown to process substrates from either terminus or from internal sites [74]. To ensure that effects would be visible regardless of the direction of processing, we included *Renilla* luciferase, which misfolds and aggregates upon heat shock and is reactivated by Hsp104 (S4A Fig), in the reporter. In this system, the N domain of Sup35 (amino acids 1–123) and its sequence variants are inserted between the two luciferase proteins (Fig 8A). Importantly, both the levels of reporter protein and activity in the absence of heat shock were identical for all of the variants studied, indicating similar efficiencies of protein maturation and stability (S4B and S4C Fig).

Using a microfluidics chamber, we monitored the relative rate of recovery of reporters containing the R1-4, ΔRPR, R2E2 and wildtype Sup35 N domains or lacking a Sup35 N insertion at 30°C in the presence of cycloheximide following a sublethal heat shock at 40°C. For each strain, the reporter protein coalesced into foci upon heat shock and resolved in an Hsp104-dependent manner (Fig 8B and S4D and S5A Figs).

The addition of the Sup35N fragment reduced the rate at which foci were resolved (S5B Fig) but did not decrease the amount of bound Hsp104 (S5C Fig), suggesting that Hsp104 binding was not limiting for substrate resolution. Consistent with this idea, the RPR, which promotes initial chaperone binding to Sup35 but becomes unnecessary in the context of a misfolded protein capable of independently recruiting chaperones (Figs 3E, 7B and 7C, S2D Fig), can be removed from the reporter without altering the rate of its resolution in comparison with the reporter containing the intact N region (Fig 8B). In contrast, deletion of the repeats (R1-4), which shifts the amyloid size to larger complexes consistent with a processing defect (Fig 3D), resolved foci more slowly than wildtype (Fig 8B), and expansion of the repeats (R2E2), which shifts the amyloid size to smaller complexes consistent with enhanced processing (Fig 3A), resolved foci faster than wildtype (Fig 8B). Thus, our observations, together, are consistent with the idea that the RPR promotes chaperone binding and that the repeats promote substrate processing.

## Discussion

While the oligopeptide repeat region of Sup35 had been previously implicated in “prion maintenance” [16], the precise mechanism by which it contributed to this process was not understood. Through detailed analyses, we have separated the oligopeptide repeat region into two elements: the repeats themselves and a repeat proximal region. We uncovered no significant contribution of either region to Sup35 conversion efficiency, aggregate kinetic stability, or chaperone levels. While we cannot rule out minor changes in these attributes outside the limit of detection for our assays, our studies suggest that the dominant role of these regions is to promote Sup35 amyloid fragmentation by molecular chaperones. This event has been previously shown to be essential for prion propagation *in vivo*, necessary to create sufficient templates to direct the conversion of soluble Sup35 to the amyloid state and to be transmitted to daughter cells upon division [40,48]. Importantly, the two sequence elements appear to mediate functional interaction of Sup35 amyloid with the chaperone machinery, through two separate activities. The repeat proximal region appears to mediate the binding of chaperones to Sup35 amyloid, while the repeats themselves appear to promote efficient chaperone processing of these aggregates.

The RPR had been previously identified as a functional element in various *in vitro* studies of Sup35 amyloid. This asparagine-rich region is predicted to be amyloidogenic on its own [75], and it is partially protected from solvent exchange and labeling in Sup35 amyloid [37,38]. In addition, residues in the RPR have been shown to be in close proximity in neighboring molecules in the Sup35 amyloid [38,76] and to be able to capture soluble Sup35 *in vitro* [39]. Finally, the RPR contains a predicted binding site for Hsp70 [77] and binds to Hsp104 *in vitro* [78,79]. Nevertheless, deletion of the RPR alone had a mild phenotypic effect on [*PSI*^*+*^]-dependent stop codon read-through *in vivo* and was not further characterized [17]. But, the synergistic effect of the RPR and the repeats on fragmentation efficiency and their inadvertent linkage in previous studies, led this element to be overlooked in favor of the repeats. Our studies now indicate that deletion of the RPR has a stronger effect on Sup35 amyloid dynamics than deletion of a single repeat (Figs 1B, 3A, 3B, 4A and 4B) and that both elements contribute separate activities to the efficiency of amyloid fragmentation and thereby prion propagation *in vivo*.

Previous studies have suggested that the amino acid composition of the Sup35 oligopeptide repeats, rather than their primary sequence *per se*, was the dominant contributor to prion propagation [80]. In these studies, Sup35 mutants were generated by scrambling the sequence of the repeat domain but leaving the amino acid composition intact. The fact that this sequence can be scrambled and still support [*PSI*^*+*^] propagation, albeit to varying efficiencies [80,81], is inconsistent with the region functioning as a primary binding site for molecular chaperones [16]. However, the potential role that we have uncovered for the repeats in substrate processing can be explained as a primary sequence independent event.

Hsp104 functions as a hexamer containing a central pore, through which substrates are threaded and unfolded. Within this pore, conformational changes in flexible aromatic residues provide the power stroke to drive substrate processing [82]. It is tempting to speculate that the low sequence complexity of the repeats affects substrate threading, and thereby amyloid fragmentation, by providing few architectural elements with which Hsp104 can interact. In this scenario, Hsp104 exerts an unfolding force as it processes Sup35, but it would disengage once it reached the repeats. Because Sup35 remains aggregated, Hsp104 would iteratively attempt to resolve these complexes, providing additional force that would ultimately lead to fragmentation. But, this outcome is also likely to be promoted by the inherent folding rate of the oligopeptide repeat region. Indeed, the processing of substrates by AAA+ ATPases in other systems has been shown to be a competition between substrate unfolding by the enzyme and its ability to refold in between each power stroke, with fast refolding requiring additional rounds of engagement [83]. Consistent with this idea, R2E2 amyloid fibers, which have a higher fragmentation rate *in vivo*, are able to quickly refold following mechanical unfolding *in vitro*, whereas repeat deletion (RΔ2–5) fibers cannot [84], differences which are likely to promote additional rounds of engagement with Hsp104 for the former but not the latter substrate.

The idea that the oligopeptide repeats promote substrate processing is also consistent with the existence of stop-transfer sequences in the substrates of other AAA+ ATPases: ClpXP and the proteasome. For example, polyQ sequences have been demonstrated to decrease the processivity of the proteasome in a length dependent manner [85–87]. In addition, the transcription factors NF-κB in higher eukaryotes and cubitus interruptus (Ci) in Drosophila both exist as full-length inactive precursors in the cytosol. In response to the appropriate signals, these precursors are then partially degraded by the proteasome to produce active truncated products, which then translocate to the nucleus and activate target gene transcription [88,89]. The partial degradation of these proteins is dependent on a stretch of simple sequence. When the proteasome reaches the simple sequence, the lack of architectural features reduces the amount of force it can exert to thread the substrate. This reduced force, combined with the nearby tightly folded domain, results in stalling of the proteasome and the release of a partially degraded product [90,91]. A similar mechanism has been proposed to explain the activation of the yeast transcription factors Spt23 and Mga2 as well as the immune evasion of the EBNA1 protein of the Epstein-Barr virus [92,93].

As an alternative scenario, specific amino acid residue(s) within the repeats may directly promote fragmentation efficiency. For example, the repeats contain tyrosine residues, which may promote interaction with the aromatic residues in the Hsp104 pore. Consistent with this idea, addition of tyrosine residues to a polyQ protein, reduces the number of Q residues required for the formation of SDS-resistant amyloids *in vivo* [94]. Importantly, this addition of tyrosines is also associated with a shift in the steady-state size distribution of these amyloids to smaller complexes, suggesting that the tyrosines facilitate fragmentation and thereby accumulation of amyloid [94]. In this model, the requirement for a minimum number of repeats would be interpreted as a threshold of tyrosine residues necessary to promote efficient fragmentation. Consistent with this idea, replacement of the tyrosines in repeats 3, 4 and 5 with non-aromatic residues leads to [*PSI*^*+*^] loss *in vivo*, although progressive effects and the mechanism by which this loss occurs were not assessed [95].

Interestingly, the number of repeats is generally conserved across a variety of amyloids. In all yeast species in which the Sup35 homologue has been shown to be capable of forming a prion in the *S*. *cerevisiae* cytoplasm, the Sup35 protein contains between five and six repeats [17,96,97]. Similarly, PrP contains five copies of an octarepeat [26,27], and bacterial functional amyloids maintain similar numbers of repeated elements, with CsgA containing 5 copies of a hexapeptide repeat and FapC containing three copies of a repeat [98,99]. One possible explanation for this similarity of repeat number across diverse species and proteins is evolutionary optimization to allow maintenance of both the amyloid and non-amyloid states. Our studies suggest that fragmentation efficiency is the mechanism underlying the contributions of repeated elements to these transitions.

While a mammalian AAA+ ATPase responsible for PrP amyloid fragmentation *in vivo* has not been identified, mathematical models suggest that the kinetics of disease progression can only be explained with a fragmentation event [100,101]. The ability of PrP repeats to functionally substitute for those of Sup35 in [*PSI*^*+*^] maintenance suggests that they could play a similar role in mammals. Recent studies have identified potential candidates for this function, including the AAA+ ATPase RuvbL [102] or the combination of Hsp70, DNAJB1, and Hsp110 [103], although their contributions to mammalian prion propagation have not yet been addressed.

Together, our studies suggest specific roles for amino acid sequence and composition biases in the propagation of a prion *in vivo*. Beyond conferring amyloid propensity, these characteristics can mediate essential functional yet mechanistically distinct interactions with the cellular chaperone machinery to promote the appearance and maintenance of alternative, self-replicating conformers *in vivo*. Their evolutionary conservation suggests a selection for these functions [27,35], and their impact on the phenotypic consequences of amyloid suggests that they may represent unique therapeutic targets.

## Methods

### Plasmids

All plasmids used in this study are listed in S3 Table; all oligos used in this study are listed in S4 Table. All plasmids generated by PCR were confirmed by sequencing.

#### Repeat deletion plasmids

Repeat deletions R1-2 (SB803), R1-3 (SB804), R1-4 (SB775), R1-5 (SB776), and ΔRPR (SB777) were constructed by amplification of a *Bam*HI/*Eco*RV Sup35 fragment with primers 5BamHISup35 and 3R1-2EcoRV, 3R1-3EcoRV, 3R1-4EcoRV, 3R1-5EcoRV, and 3Sup35R1-6EcoRV respectively and using SLL6686 as a template. *Bam*HI/*Eco*RV fragments were inserted into SLL6686. R1-4ΔRPR (SB549) and R1-5ΔRPR (SB550) were constructed as previously described [17]. R2E2Δ4–5 (SB1008) was synthesized as a *Bam*HI/*Bse*RI fragment (Genewiz) and inserted into SLL6686.

#### Repeat expansion plasmids

R2E1 (SB787) was constructed by annealing oligos R2E1 insertF and R2E1 insertR, and insertion of the resulting product into the *Bst*XI site of SLL6686. R2E2 (SB859) was constructed by QuikChange (Stratagene) of SB787 with R2 *Bst*XI QCF and R2 BstXI QCR according to the manufacturer’s instructions, followed by insertion of annealed oligos R2E1 insertF and R2E1 insertR into the remaining *Bst*XI site. SB883 and SB884 were constructed by insertion of the R2E1 and R2E2 ORFs, respectively, as an *Eco*RI/*Sac*I fragment into pRS303-P_SUP35_.

#### HA-tagged constructs

NM-3HA constructs containing R1-5, ΔRPR, and R2E1 were constructing by inserting a *Pst*I/*Msc*I fragment from SB776, SB777, or SB787 respectively into SB653. NM-3HA-C constructs containing RWT, R1-5, and ΔRPR were constructed by insertion of the *Bgl*II/*Sac*I fragment from pRS426-P_Sup35_NM-3HA-C (gift from J. Weissman) into SB653, SB1040, SB1041, and SB1042 respectively.

#### Conversion read-through plasmid

pRS304-P_GPD_GST(UGA)YFP-NLS was constructed by replacing the DsRedNLS open reading frame in SB531 using *Eco*RI and *Cla*I sites. YFP-NLS was generated as a PCR fragment using the primers 5EcoRI Citrine and 3ClaI CitrineNLS and pKT140 (Addgene) as a template.

#### Dual Luciferase constructs

SB973, SB994, SB975, SB976, and SB985 contain the GPD promoter driving firefly luciferase-Sup35N-Renilla Luciferase-GFPGAr. A three-repeat glycine-serine linker separates each of the ORFs. ORFs for Firefly luciferase (*Xba*I/*Bam*HI PCR fragment generated using 5XbaI Firefly and 3BamHI Firefly), Sup35N (*Bam*HI/*Bam*HI PCR fragment generated using 5BamHIGS3 Sup35N and 3BamHI Sup35N), Renilla luciferase (*Bam*HI/*Eco*RI PCR fragment generated using 5BamHIGS3 Renilla and 3 EcoRI Renilla), and GFPGAr (*Eco*RI/*Xho*I PCR fragment generated using 5EcoRIGS3 GFP and 3XhoIGAr GFP), were inserted into SB237. For Sup35N Rwt, R1-4, R2E2, and ΔRPR, PCR templates were SLL6686, SB775, SB859, and SB777 respectively. SB986 contains Renilla Luciferase-GFP, with the open reading frames separated by a three-repeat glycine-serine linker. Renilla luciferase (*Xba*I/*Eco*RI PCR fragment generated using 5XbaI Renilla and 3EcoRI Renilla), and GFP-GAr (*Eco*RI/*Xho*I PCR fragment generated using 5EcoRIGS3 GFP and 3XhoIGAr GFP) were inserted into SB237 (pRS305-P_GPD_).

### Strain Construction

All strains used in this study are listed in S5 Table and are strong [*PSI*^*+*^] derivatives of 74-D694 [104].

#### Repeat Variant Strains

SY2072 (R1-2), SY2073 (R1-3), SY2057 (R1-4), SY 2022 (R1-5), SY1629 (R1-4ΔRPR), SY1633 (R1-5ΔRPR), SY2023 (ΔRPR), and SY2808 (R2E2Δ4–5) were constructed by integration of *Mlu*I-digested SB803, SB804, SB775, SB776, SB549, SB550, SB777, and SB1008 respectively, selection on SD-Ura, followed by counter selection on 5-FOA. Strains were screened for repeat number by PCR using 5Sup35Nrepck and 3Sup35repck, and confirmed by sequencing and for endogenous levels of Sup35 expression. SY2247 (R2E1) was constructed by integration of *Mlu*I-digested SB787, selection on SD-Ura, and counter-selection on 5-FOA, followed by integration of *Afe*I-digested SB883 and selection on SD-His. Strains were screened for repeat number by PCR using 5Sup35Nrepck and 3Sup35repck, and confirmed by sequencing and for endogenous levels of Sup35 expression. SY2300 (R2E2) was constructed by integration of *Mlu*I-digested SB859, selection on SD-Ura, and counter selection on 5-FOA, followed by integration of *Afe*I-digested SB883, followed by selection on SD-His. To obtain endogenous levels of Sup35 expression, the promoter at the endogenous locus was replaced with P_MFA1_ by integration of a PCR-generated cassette, using F4-PSup35 and R2-PMFAI primers, with SB526 (pFA6a-KanMX6-P_MFA1_) as a template, and selection on YPD+G418. Strains were screened for repeat number by PCR using 5Sup35Nrepck and 3Sup35repck, and confirmed by sequencing and for endogenous levels of Sup35 expression. [*psi*^-^] versions of repeat variant strains were made by curing the [*PSI*^*+*^] versions on YPD + 3mM guanidine HCl (GdnHCl), and selection for colony color. SY2466 and SY2467 were constructed by integration of *PpuM*I digested SLL6682 into SLL2606, and SY2057 respectively, selection on SD-Ura, and screening by colony color. [*PSI*^*+*^] was confirmed by SDD-AGE. SY3007, SY3008, SY3009, and SY3010 were constructed by integration of *PpuM*I-digested SB653, SB1040, SB1041, and SB1042 into SY2606, SY2022, SY2023, and SY2247 respectively. SY3159, and SY3164 were constructed by integration of *PpuM*I-digested SB1091, and SB1093 into SY2606, and SY2023 respectively. Hsp104Y662F point mutation was introduced into SLL3250 by *delitto perfetto* [105] to generate SY2991, which was subsequently mated to repeat variant strains SLL2606, SY2022, SY2023, and SY2247 and tetrads were dissected to generate SY3001, SY3002, SY3004, and SY3005 respectively.

#### Conversion strains

SY2393 was constructed by integration of *PpuM*I-digested SB910 in SLL3250, selection on SD-Trp, and screening by fluorescence, followed by prion curing by 3mM guanidine HCl (GdnHCl) treatment. SY2461, SY2463, and SY2465 were constructed by mating SY2393 to SY2215, SY2212, and SY2302 respectively, followed by sporulation, dissection and selection on SD–Trp. Repeat number in tetrads was screened by PCR using primers 5Sup35Nrepck and 3Sup35Nrepck.

#### Luciferase strains

SY2597, SY2603, SY2637, SY2640, SY2666, and SY2694 were constructed by integration of *Stu*I-digested SB973, SB994, SB975, SB976, SB985, and SB986 and respectively. Strains were screened by luminescence and fluorescence. Hsp104 disruptions SY2620, SY2625, SY2651, SY2654, SY2679, and SY2703 were constructed by integration of a *Pvu*I/*Bam*HI digested fragment of pYABL5 (gift from S. Lindquist) into SY2597, SY2603, SY2637, SY2640, SY2666, and SY2694 respectively and selection on SD-Leu.

### Growth conditions

All strains were grown in rich medium supplemented with 3mM adenine (YPAD), unless otherwise specified. Cultures were grown in a shaking incubator at 30°C and maintained at an OD_600_ of less than 0.5 for at least 10 doublings to ensure exponential growth.

### Protein Analysis

SDS-PAGE and quantitative immunoblotting were performed as previously described [106]. Semi-Denaturing Detergent Agarose Electrophoresis (SDD-AGE) was performed as previously described [51].

### Propagon Counts

The number of propagons per cell was determined by an *in vivo* colony-based dilution assay, as previously described [52]. For propagon amplification experiments, cultures were first grown in YPAD + 3mM GdnHCl for 12 hours. Then, cells were pelleted, resuspended in YPAD to an OD_600_ of 0.1, and grown at 30°C. Propagon counts were then performed at the indicated timepoints.

### Conversion Assay

Cultures were grown in SD+2.5mM adenine overnight, collected by centrifugation and incubated in medium conditioned by cells of the opposite mating type for one hour. Equal OD_600_ equivalents of each mating partner were then mixed and incubated on solid SD + 2.5mM adenine and allowed to mate for 4 hours at 30°C (where indicated, mating took place on solid SD + 2.5mM adenine + 3mM GdnHCl). Cells were then resuspended in SD + 2.5mM adenine and transferred to microscope slides for imaging.

### Imaging

Imaging was performed in complete minimal medium supplemented with 2.5mM adenine and 2% glucose. Static images were obtained on a Zeiss Axio Imager M2 fluorescent light microscope with a 100x objective. Microfluidics were performed on a Zeiss Axio Observer Z1 using a CellAsics microfluidics plate with temperature controls and media flow of 2 psi on a Y0C4 yeast perfusion plate (channel size 3.5–5μm). Fluorescence intensity was analyzed using the Zen software package (Zeiss, Germany).

### Immunocapture

For NM-HA and NM-HA-C immunocapture, native lysates were prepared as described [61], and immunocapture was performed using anti-HA magnetic beads or anti-Myc magnetic beads (Thermo Scientific Pierce). Co-captured proteins were resolved by SDS-PAGE and analyzed by western blotting for Sup35, HA (Roche), Hsp104 (Abcam), Ssa1 (gift from E. Craig), and Sis1 (gift from M. Tuite). The amount of Sup35 and NM-HA or NM-HA-C captured was adjusted to reflect only the amount of Sup35 proteins present in aggregates in each strain, as determined by incubating lysates at 53°C and 100°C in the presence of SDS and resolving the protein on an SDS-PAGE gel. The percentage of protein in aggregates was then calculated as the fraction of Sup35 that did not enter the gel at 53°C. The amount of each chaperone that was co-captured was then compared to the amount of captured aggregated Sup35.

For Hsp104 binding to luciferase reporters, cells were incubated at 37°C for 30 minutes followed by 40°C for 35 minutes, with the addition of cycloheximide to 100μg/mL for the last 10 minutes. Cell lysates were prepared, and immunocapture was performed as described [61], except 600mM NaCl was used in lysis and wash buffers. Co-captured proteins were separated by SDS-PAGE and analyzed by western blotting for Firefly luciferase (Sigma) and Hsp104 (Abcam).

### Luciferase Recovery

Cultures were grown to an OD_600_ of 0.1 at 30°C, then incubated at 37°C for 30 minutes to induce chaperone expression. Cultures were then incubated at 40°C for 25 minutes, followed by addition of cycloheximide to 100μg/mL, and returned to 40°C for 10 minutes, followed by recovery at 30°C. Cells were imaged at the indicated timepoints.

## Supporting Information

S1 Fig Sup35 expression levels are similar in all sequence variant strains. **A.** Lysates from R1-2 (SY2072), R1-3 (SY2073), R1-4ΔRPR (SY1629), R1-4 (SY2057), R1-5ΔRPR (SY1633), R1-5 (SY2022), ΔRPR (SY2023), R2E1 (SY2247), R2E2 (SY2300), and R2E2Δ4–5 (SY2808) were analyzed by SDS-PAGE and quantitative immunoblotting for Sup35. Bars represent means; error bars represent standard deviations. n≥3. **B.** R1-2 (SY2072), R1-3 (SY2073) were spotted onto rich medium (YPD) and medium lacking adenine (-Ade) to analyze the [*PSI*^*+*^] phenotype. Wildtype [*PSI*^*+*^] (SLL2606) is shown as a control. **C.** SDD-AGE was performed on wildtype [*PSI*^*+*^] (SLL2606), R1-2 (SY2072), and R1-3 (SY2073) lysates, followed by immunoblotting for Sup35. Panels shown are non-consecutive lanes run on the same gel. **D.** SDD-AGE was performed on R1-4 (SY2057) and R1-4ΔRPR (SY1629) lysates, followed by immunoblotting for Sup35. **E**. SDD-AGE was performed on wildtype [*PSI*^*+*^] (SLL2606), R2E2Δ4–5 (SY2808), and R2E2 (SY2247) lysates, followed by immunoblotting for Sup35.
(TIF)

S2 Fig Characterization of strains used for immunocapture. **A.** Lysates from wildtype (SY3007), R1-5 (SY3008), R2E1 (SY3010), and ΔRPR (SY3009) strains expressing NM-HA were analyzed by SDS-PAGE and quantitative immunoblotting for HA (Bars represent means, error bars represent standard deviations, n≥3). **B.** SDD-AGE was performed on lysates from wildtype (SY3007), R1-5 (SY3008), R2E1 (SY3010) and ΔRPR (SY3009) strains expressing full-length Sup35 (FL) and NM-HA and immunoblotted for Sup35 n≥3. **C.** Lysates from wildtype (SY3159), and ΔRPR (SY3164) strains expressing full-length Sup35 (FL) and NM-HA-C were analyzed by SDS-PAGE and quantitative immunoblotting for HA (Bars represent means, error bars represent standard deviations, n≥3). **D.** SDD-AGE was performed on cell lysates from wildtype (SY3159) and ΔRPR (SY3164), strains expressing full-length Sup35 (FL) and NM-HA-C followed by immunoblotting for Sup35.
(TIF)

S3 Fig Hsp104, Ssa1, and Sis1 bind to Sup35 containing repeat or RPR variants. **A.** Representative gel from experiment quantified in Fig 7A. Aggregates in wildtype (SY3007), R1-5 (SY3008), R2E1 (SY3010), and ΔRPR (SY3009) strains expressing NM-HA and full length Sup35 were immunocaptured using anti-HA magnetic beads and separated by SDS-PAGE and the amount of bound chaperone proteins was determined by immunoblotting. **B.** Representative gel from experiment quantified in Fig 7B. Aggregates in wildtype (SY3159) and ΔRPR (SY3164) strains expressing NM-HA-C and full length (untagged) Sup35 were immunocaptured with anti-HA magnetic beads and separated by SDS-PAGE, and the amount of bound Hsp104, Ssa1, and Sis1 was determined by immunoblotting. Panels shown are non-consecutive lanes run on the same gel. **C.** Wildtype (SY3159) strains expressing NM-HA-C and full-length (untagged) Sup35 were treated as in A, and the amount of Hsp104, Ssa1, and Sis1 bound was determined by immunoblotting. Panels shown are non-consecutive lanes run on the same gel.
(TIF)

S4 Fig Recovery of dual luciferase constructs following heat shock is dependent on Hsp104. **A.** Strains containing *Renilla* luciferase-GFP with or without an Hsp104 disruption (SY2694 and SY2703, respectively) were grown in rich medium, heat shocked at 37°C for 30 min followed by 40°C for 35 minutes with cycloheximide added for the last 10 minutes, and allowed to recover at 30°C in the presence of cycloheximide. *Renilla* luciferase activity was measured at the indicated time points. n = 3, data represent means; error bars indicate standard deviations. **B.** Lysates from strains containing Firefly-Sup35N-*Renilla*-GFP reporters were analyzed by SDS-PAGE and quantitative immunoblotting for GFP. n = 3, bars represent means; error bars represent standard deviations. **C.** The levels of firefly and *Renilla* luciferase activity were determined in strains expressing the indicated reporters. n = 3, bars represent means; error bars represent standard deviations. **D.** Strains containing firefly luciferase-Sup35N(RV)-*Renilla* luciferase-GFP with or without an Hsp104 disruption were treated as in A, and imaged at the indicated time points. Representative images are shown, scale bar = 5μm.
(TIF)

S5 Fig **Insertion of Sup35N in dual luciferase reporter delays substrate processing A.** A [*psi*^-^] strain containing firefly luciferase-Renilla luciferase-GFP with (SY2620) or without (SY2603) an Hsp104 disruption were grown in rich medium, heat shocked at 37°C for 30 min followed by 40°C for 35 minutes with cycloheximide added for the last 10 minutes, and allowed to recover at 30°C in the presence of cycloheximide, and imaged at the indicated time points. Representative images are shown. Scale bar = 2μm. **B.** The indicated strains expressing the dual luciferase reporter without (SY2597) and with (SY2603) Sup35N were incubated at 37°C for 30 minutes, followed by 40°C for 35 minutes, with cycloheximide added for the last 10 minutes, in a microfluidics chamber. Cells were then allowed to recover at 30°C in the presence of cycloheximide, and images were taken at the indicated time points. At each time point, the percentage of cells containing GFP foci was determined. n≥38. **C.** Dual luciferase reporter fusion proteins without (SY2597) and with (SY2603) Sup35N were immunocaptured, separated by SDS-PAGE and the amount of bound Hsp104 was quantified by western blot. n = 6, bars represent means; error bars represent standard deviations.
(PDF)

S1 Text Mathematical Modeling (DOCX)

S1 Table Significantly Different Levels of Soluble Sup35% (DOCX)

S2 Table Significantly Different R_0_ Values (DOCX)

S3 Table Plasmids (DOCX)

S4 Table Oligonucleotides (DOCX)

S5 Table Yeast Strains (DOCX)

## References

[1] Knowles TPJ, Vendruscolo M, Dobson CM. The amyloid state and its association with protein misfolding diseases. Nature Rev Mol Cell Biol. 2014;15: 384–396. 10.1038/nrm3810 24854788

[2] Tuite MF, Serio TR. The prion hypothesis: from biological anomaly to basic regulatory mechanism. Nature Rev Mol Cell Biol. 2010;11: 823–833. 10.1038/nrm3007 21081963 PMC3003427

[3] March ZM, King OD, Shorter J. Prion-like domains as epigenetic regulators, scaffolds for subcellular organization, and drivers of neurodegenerative disease. Brain Res. 2016. 10.1016/j.brainres.2016.02.037 PMC5003744 26996412

[4] Si K, Choi YB, White-Grindley E, Majumdar A, Kandel ER. Aplysia CPEB can form prion-like multimers in sensory neurons that contribute to long-term facilitation. Cell. 2010;140: 421–435. 10.1016/j.cell.2010.01.008 20144764

[5] Cai X, Chen J, Xu H, Liu S, Jiang Q-X, Halfmann R, et al Prion-like polymerization underlies signal transduction in antiviral immune defense and inflammasome activation. Cell. 2014;156: 1207–1222. 10.1016/j.cell.2014.01.063 24630723 PMC4034535

[6] Fowler DM, Koulov AV, Alory-Jost C, Marks MS, Balch WE, Kelly JW. Functional amyloid formation within mammalian tissue. PLOS Biol. 2006;4: e6 10.1371/journal.pbio.0040006 16300414 PMC1288039

[7] Cascarina SM, Ross ED. Yeast prions and human prion-like proteins: sequence features and prediction methods. Cell Mol Life Sci. 2014;71: 2047–2063. 10.1007/s00018-013-1543-6 24390581 PMC4024371

[8] Perutz MF, Johnson T, Suzuki M, Finch JT. Glutamine repeats as polar zippers: their possible role in inherited neurodegenerative diseases. Proc Natl Acad Sci U S A. 1994;91: 5355–5358. 8202492 10.1073/pnas.91.12.5355 PMC43993

[9] Sunde M, Blake C. The structure of amyloid fibrils by electron microscopy and X-ray diffraction. Adv Protein Chem. 1997;50: 123–159. 9338080 10.1016/s0065-3233(08)60320-4

[10] Eisenberg D, Jucker M. The amyloid state of proteins in human diseases. Cell. 2012;148: 1188–1203. 10.1016/j.cell.2012.02.022 22424229 PMC3353745

[11] López de la Paz M, Serrano L. Sequence determinants of amyloid fibril formation. Proc Natl Acad Sci U S A. 2004;101: 87–92. 10.1073/pnas.2634884100 14691246 PMC314143

[12] Alberti S, Halfmann R, King O, Kapila A, Lindquist S. A systematic survey identifies prions and illuminates sequence features of prionogenic proteins. Cell. 2009;137: 146–158. 10.1016/j.cell.2009.02.044 19345193 PMC2683788

[13] Tuite MF, Cox BS. Propagation of yeast prions. Nature Rev Mol Cell Biol. 2003;4: 878–890. 10.1038/nrm1247 14625537

[14] Chien P, Weissman JS, DePace AH. Emerging principles of conformation-based prion inheritance. Ann Rev Biochem. 2004;73: 617–656. 10.1146/annurev.biochem.72.121801.161837 15189155

[15] Tessier PM, Lindquist S. Unraveling infectious structures, strain variants and species barriers for the yeast prion [*PSI*^*+*^]. Nature Struct Mol Biol. 2009;16: 598–605. 10.1038/nsmb.1617 19491937 PMC4502417

[16] Osherovich LZ, Cox BS, Tuite MF, Weissman JS. Dissection and design of yeast prions. PLOS Biol. 2004;2: E86 10.1371/journal.pbio.0020086 15045026 PMC374241

[17] Parham SN, Resende CG, Tuite MF. Oligopeptide repeats in the yeast protein Sup35p stabilize intermolecular prion interactions. EMBO J. 2001;20: 2111–9. 10.1093/emboj/20.9.2111 11331577 PMC125439

[18] Ter-Avanesyan MD, Dagkesamanskaya AR, Kushnirov VV, Smirnov VN. The SUP35 omnipotent suppressor gene is involved in the maintenance of the non-Mendelian determinant [*PSI*^*+*^] in the yeast *Saccharomyces cerevisiae*. Genetics. 1994;137: 671–676. 8088512 10.1093/genetics/137.3.671 PMC1206026

[19] Young C, Cox BS. Extrachromosomal Elements in A Super-Suppression System of Yeast. I. A Nuclear Gene Controlling The Inheritance of the Extrachromosomal Elements. Heredity. 1971;26: 413–422.

[20] Doel SM, McCready SJ, Nierras CR, Cox BS. The dominant PNM2- mutation which eliminates the psi factor of Saccharomyces cerevisiae is the result of a missense mutation in the SUP35 gene. Genetics. 1994; 137:659–70. 8088511 10.1093/genetics/137.3.659 PMC1206025

[21] Liu JJ, Lindquist S. Oligopeptide-repeat expansions modulate “protein-only” inheritance in yeast. Nature. 1999;400: 573–576. 10.1038/23048 10448860

[22] Crist CG, Nakayashiki T, Kurahashi H, Nakamura Y. [*PHI*^*+*^], a novel Sup35-prion variant propagated with non-Gln/Asn oligopeptide repeats in the absence of the chaperone protein Hsp104. Genes Cells. 2003;8: 603–618. 12839621 10.1046/j.1365-2443.2003.00661.x

[23] Bondarev SA, Shchepachev VV, Kajava AV, Zhouravleva GA. Effect of charged residues in the N-domain of Sup35 protein on prion [*PSI^+^*] stability and propagation. J Biol Chem. 2013;288: 28503–28513. 10.1074/jbc.M113.471805 23965990 PMC3789951

[24] King C-Y. Supporting the structural basis of prion strains: induction and identification of [*PSI*] variants. J Mol Biol. 2001;307: 1247–1260. 10.1006/jmbi.2001.4542 11292339

[25] Chang H-Y, Lin J-Y, Lee H-C, Wang H-L, King C-Y. Strain-specific sequences required for yeast [*PSI*^*+*^] prion propagation. Proc Natl Acad Sci U S A. 2008;105: 13345–13350. 10.1073/pnas.0802215105 18757753 PMC2533192

[26] Kretzschmar HA, Stowring LE, Westaway D, Stubblebine WH, Prusiner SB, DeArmond SJ. Molecular cloning of a human prion protein cDNA. DNA. 1986;5: 315–324. 3755672 10.1089/dna.1986.5.315

[27] van Rheede T, Smolenaars MMW, Madsen O, de Jong WW. Molecular evolution of the mammalian prion protein. Mol Biol Evol. 2003;20: 111–121. 12519913 10.1093/molbev/msg014

[28] Pietrini V, Puoti G, Limido L, Rossi G, Di Fede G, Giaccone G, et al Creutzfeldt-Jakob disease with a novel extra-repeat insertional mutation in the PRNP gene. Neurol. 2003;61: 1288–1291. 14610142 10.1212/01.wnl.0000092017.74772.ca

[29] Kumar N, Boeve BF, Boot BP, Orr CF, Duffy J, Woodruff BK, et al Clinical characterization of a kindred with a novel 12-octapeptide repeat insertion in the prion protein gene. Arch Neurol. 2011;68: 1165–1170. 10.1001/archneurol.2011.187 21911696 PMC3326586

[30] Mead S, Poulter M, Beck J, Webb TEF, Campbell TA, Linehan JM, et al Inherited prion disease with six octapeptide repeat insertional mutation—molecular analysis of phenotypic heterogeneity. Brain. 2006;129: 2297–2317. 10.1093/brain/awl226 16923955

[31] Mead S, Webb TEF, Campbell TA, Beck J, Linehan JM, Rutherfoord S, et al Inherited prion disease with 5-OPRI: phenotype modification by repeat length and codon 129. Neurol. 2007;69: 730–738. 10.1212/01.wnl.0000267642.41594.9d 17709704

[32] Croes EA, Theuns J, Houwing-Duistermaat JJ, Dermaut B, Sleegers K, Roks G, et al Octapeptide repeat insertions in the prion protein gene and early onset dementia. J Neurol Neurosurg Psychiatr. 2004;75: 1166–1170. 10.1136/jnnp.2003.020198 15258222 PMC1739180

[33] Tank EMH, Harris DA, Desai AA, True HL. Prion protein repeat expansion results in increased aggregation and reveals phenotypic variability. Mol Cell Biol. 2007;27: 5445–5455. 10.1128/MCB.02127-06 17548473 PMC1952097

[34] Dong J, Bloom JD, Goncharov V, Chattopadhyay M, Millhauser GL, Lynn DG, et al Probing the role of PrP repeats in conformational conversion and amyloid assembly of chimeric yeast prions. J Biol Chem. 2007;282: 34204–34212. 10.1074/jbc.M704952200 17893150 PMC2262835

[35] Harrison LB, Yu Z, Stajich JE, Dietrich FS, Harrison PM. Evolution of budding yeast prion-determinant sequences across diverse fungi. J Mol Biol. 2007;368: 273–282. 10.1016/j.jmb.2007.01.070 17320905

[36] Shkundina IS, Kushnirov VV, Tuite MF, Ter-Avanesian MD. The role of the N-terminal oligopeptide repeats of the yeast Sup35 prion protein in propagation and transmission of prion variants. Genetics. 2006;172: 827–835. 10.1534/genetics.105.048660 16272413 PMC1456247

[37] Toyama BH, Kelly MJS, Gross JD, Weissman JS. The structural basis of yeast prion strain variants. Nature. 2007;449: 233–237. 10.1038/nature06108 17767153

[38] Krishnan R, Lindquist SL. Structural insights into a yeast prion illuminate nucleation and strain diversity. Nature. 2005;435: 765–772. 10.1038/nature03679 15944694 PMC1405905

[39] Tessier PM, Lindquist S. Prion recognition elements govern nucleation, strain specificity and species barriers. Nature. 2007;447: 556–561. 10.1038/nature05848 17495929 PMC2144736

[40] Derdowski A, Sindi SS, Klaips CL, DiSalvo S, Serio TR. A size threshold limits prion transmission and establishes phenotypic diversity. Science 2010;330: 680–683. 10.1126/science.1197785 21030659 PMC3003433

[41] Kushnirov VV, Ter-Avanesyan MD, Surguchov AP, Smirnov VN, Inge-Vechtomov SG. Localization of possible functional domains in sup2 gene product of the yeast S*accharomyces cerevisiae*. FEBS Letters. 1987;215: 257–260. 3556215 10.1016/0014-5793(87)80157-6

[42] Kushnirov VV, Ter-Avanesyan MD, Telckov MV, Surguchov AP, Smirnov VN, Inge-Vechtomov SG. Nucleotide sequence of the SUP2 (SUP35) gene of *Saccharomyces cerevisiae*. Gene. 1988;66: 45–54. 3047009 10.1016/0378-1119(88)90223-5

[43] Chernoff YO, Inge-Vechtomov SG, Derkach IL, Ptyushkina MV, Tarunina OV, Dagkesamanskaya AR, et al Dosage-dependent translational suppression in yeast *Saccharomyces cerevisiae*. Yeast. 1992;8: 489–499. 10.1002/yea.320080702 1523883

[44] Serio TR, Lindquist SL. [*PSI*^*+*^]: an epigenetic modulator of translation termination efficiency. Ann Rev Cell Dev Biol. 1999;15: 661–703. 10.1146/annurev.cellbio.15.1.661 10611975

[45] Derkatch IL, Chernoff YO, Kushnirov VV, Inge-Vechtomov SG, Liebman SW. Genesis and variability of [*PSI*] prion factors in *Saccharomyces cerevisiae*. Genetics. 1996;144: 1375–1386. 8978027 10.1093/genetics/144.4.1375 PMC1207691

[46] DiSalvo S, Derdowski A, Pezza JA, Serio TR. Dominant prion mutants induce curing through pathways that promote chaperone-mediated disaggregation. Nature Struct Mol Biol. 2011;18: 486–492. 10.1038/nsmb.2031 21423195 PMC3082495

[47] Satpute-Krishnan P, Serio TR. Prion protein remodelling confers an immediate phenotypic switch. Nature. 2005;437: 262–265. 10.1038/nature03981 16148935

[48] Satpute-Krishnan P, Langseth SX, Serio TR. Hsp104-dependent remodeling of prion complexes mediates protein-only inheritance. PLOS Biol. 2007;5: e24 10.1371/journal.pbio.0050024 17253904 PMC1779812

[49] Eaglestone SS, Ruddock LW, Cox BS, Tuite MF. Guanidine hydrochloride blocks a critical step in the propagation of the prion-like determinant [*PSI^+^*] of *Saccharomyces cerevisiae*. Proc Natl Acad Sci U S A. 2000;97: 240–244. 10618402 10.1073/pnas.97.1.240 PMC26647

[50] Ness F, Ferreira P, Cox BS, Tuite MF. Guanidine hydrochloride inhibits the generation of prion “seeds” but not prion protein aggregation in yeast. Mol Cell Biol. 2002;22: 5593–5605. 10.1128/MCB.22.15.5593-5605.2002 12101251 PMC133959

[51] Kryndushkin DS, Alexandrov IM, Ter-Avanesian MD, Kushnirov VV. Yeast [*PSI*^*+*^] prion aggregates are formed by small Sup35 polymers fragmented by Hsp104. J Biol Chem. 2003;278: 49636–49643. 10.1074/jbc.M307996200 14507919

[52] Cox B, Ness F, Tuite M. Analysis of the generation and segregation of propagons: entities that propagate the [*PSI*^*+*^] prion in yeast. Genetics. 2003;165: 23–33. 14504215 10.1093/genetics/165.1.23 PMC1462756

[53] Holmes WM, Klaips CL, Serio TR. Defining the limits: Protein aggregation and toxicity in vivo. Crit Rev Biochem Mol Biol. 2014; 49: 1–10. 10.3109/10409238.2014.914151 24766537 PMC4238936

[54] Vishveshwara N, Bradley ME, Liebman SW. Sequestration of essential proteins causes prion associated toxicity in yeast. Mol Microbiol. 2009; 73: 1101–1114. 10.1111/j.1365-2958.2009.06836.x 19682262 PMC2757070

[55] Tanaka M, Collins SR, Toyama BH, Weissman JS. The physical basis of how prion conformations determine strain phenotypes. Nature. 2006;442: 585–589. 10.1038/nature04922 16810177

[56] Jung G, Jones G, Masison DC. Amino acid residue 184 of yeast Hsp104 chaperone is critical for prion-curing by guanidine, prion propagation, and thermotolerance. Proc Natl Acad Sci U S A. 2002;99: 9936–41. 10.1073/pnas.152333299 12105276 PMC126603

[57] Jung G, Masison DC. Guanidine hydrochloride inhibits Hsp104 activity *in vivo*: a possible explanation for its effect in curing yeast prions. Curr Microbiol. 2001;43: 7–10. 10.1007/s002840010251 11375656

[58] Wegrzyn RD, Bapat K, Newnam GP, Zink AD, Chernoff YO. Mechanism of prion loss after Hsp104 inactivation in yeast. Mol Cell Biol. 2001;21: 4656–4669. 10.1128/MCB.21.14.4656-4669.2001 11416143 PMC87136

[59] Lum R, Tkach JM, Vierling E, Glover JR. Evidence for an unfolding/threading mechanism for protein disaggregation by *Saccharomyces cerevisiae* Hsp104. J Biol Chem. 2004;279: 29139–29146. 10.1074/jbc.M403777200 15128736

[60] Hung G-C, Masison DC. N-terminal domain of yeast Hsp104 chaperone is dispensable for thermotolerance and prion propagation but necessary for curing prions by Hsp104 overexpression. Genetics. 2006;173: 611–620. 10.1534/genetics.106.056820 16582428 PMC1526498

[61] Klaips CL, Hochstrasser ML, Langlois CR, Serio TR. Spatial quality control bypasses cell-based limitations on proteostasis to promote prion curing. Elife. 2014;3: 1751 10.7554/eLife.04288 25490068 PMC4270096

[62] Holmes WM, Mannakee BK, Gutenkunst RN, Serio TR. Loss of amino-terminal acetylation suppresses a prion phenotype by modulating global protein folding. Nat Commun. 2014;5: 4383 10.1038/ncomms5383 25023910 PMC4140192

[63] Newnam GP, Birchmore JL, Chernoff YO. Destabilization and recovery of a yeast prion after mild heat shock. J Mol Biol. 2011 ed. 2011;408: 432–448. 10.1016/j.jmb.2011.02.034 21392508 PMC3095851

[64] Verges KJ, Smith MH, Toyama BH, Weissman JS. Strain conformation, primary structure and the propagation of the yeast prion [*PSI*^*+*^]. Nature Struct Mol Biol. 2011;18: 493–499. 10.1038/nsmb.2030 21423194 PMC3490428

[65] Alexandrov AI, Polyanskaya AB, Serpionov GV, Ter-Avanesyan MD, Kushnirov VV. The effects of amino acid composition of glutamine-rich domains on amyloid formation and fragmentation. PLOS One. 2012;7: e46458 10.1371/journal.pone.0046458 23071575 PMC3468588

[66] Derkatch IL, Bradley ME, Zhou P, Liebman SW. The PNM2 mutation in the prion protein domain of SUP35 has distinct effects on different variants of the [*PSI*^*+*^] prion in yeast. Curr Genet. 1999;35: 59–67. 10079323 10.1007/s002940050433

[67] Serio TR, Cashikar AG, Kowal AS, Sawicki GJ, Moslehi JJ, Serpell L, Arnsdorf MF, Lindquist SL. Nucleated Conformational Conversion and the Replication of Conformational Information by a Prion Determinant. Science. 2000;289: 1317–1321. 10958771 10.1126/science.289.5483.1317

[68] Chien P, Weissman JS. Conformational diversity in a yeast prion dictates its seeding specificity. Nature. 2001;410: 223–7. 10.1038/35065632 11242084

[69] Winkler J, Tyedmers J, Bukau B, Mogk A. Hsp70 targets Hsp100 chaperones to substrates for protein disaggregation and prion fragmentation. J Cell Biol. 2012;198: 387–404. 10.1083/jcb.201201074 22869599 PMC3413357

[70] Tipton KA, Verges KJ, Weissman JS. In vivo monitoring of the prion replication cycle reveals a critical role for Sis1 in delivering substrates to Hsp104. Mol Cell. 2008 ed. 2008;32: 584–591. 10.1016/j.molcel.2008.11.003 19026788 PMC2875781

[71] Higurashi T, Hines JK, Sahi C, Aron R, Craig EA. Specificity of the J-protein Sis1 in the propagation of 3 yeast prions. Proc Natl Acad Sci U S A. 2008;105: 16596–16601. 10.1073/pnas.0808934105 18955697 PMC2575465

[72] Abrams JL, Morano KA. Coupled assays for monitoring protein refolding in Saccharomyces cerevisiae. J Vis Exp. 2013;77: e50432–e50432. 10.3791/50432 23892247 PMC3732071

[73] Parsell DA, Kowal AS, Singer MA, Lindquist S. Protein disaggregation mediated by heat-shock protein Hsp104. Nature. 1994;372: 475–478. 10.1038/372475a0 7984243

[74] Koodathingal P, Jaffe NE, Kraut DA, Prakash S, Fishbain S, Herman C, et al ATP-dependent proteases differ substantially in their ability to unfold globular proteins. J Biol Chem 2009;284: 18674–18684. 10.1074/jbc.M900783200 19383601 PMC2707231

[75] Pastor MT, Esteras-Chopo A, Serrano L. Hacking the code of amyloid formation: the amyloid stretch hypothesis. Prion. 2007;1: 9–14. 19164912 10.4161/pri.1.1.4100 PMC2633701

[76] Shewmaker F, Wickner RB, Tycko R. Amyloid of the prion domain of Sup35p has an in-register parallel beta-sheet structure. P Proc Natl Acad Sci U S A. 2006;103: 19754–19759. 10.1073/pnas.0609638103 17170131 PMC1750918

[77] Van Durme J, Maurer-Stroh S, Gallardo R, Wilkinson H, Rousseau F, Schymkowitz J. Accurate prediction of DnaK-peptide binding via homology modelling and experimental data. Tramontano A, editor. PLOS Comput Biol. 2009;5: e1000475 10.1371/journal.pcbi.1000475 19696878 PMC2717214

[78] Sweeny EA, Jackrel ME, Go MS, Sochor MA, Razzo BM, DeSantis ME, et al The Hsp104 N-terminal domain enables disaggregase plasticity and potentiation. Mol Cell. 2015;57: 836–849. 10.1016/j.molcel.2014.12.021 25620563 PMC4623595

[79] Helsen CW, Glover JR. Insight into molecular basis of curing of [PSI+] prion by overexpression of 104-kDa heat shock protein (Hsp104). J Biol Chem. 2012;287: 542–556. 10.1074/jbc.M111.302869 22081611 PMC3249108

[80] Toombs JA, Liss NM, Cobble KR, Ben-Musa Z, Ross ED. [*PSI*^*+*^] maintenance is dependent on the composition, not primary sequence, of the oligopeptide repeat domain. PLOS One. 2011;6: e21953 10.1371/journal.pone.0021953 21760933 PMC3132755

[81] Ross ED, Edskes HK, Terry MJ, Wickner RB. Primary sequence independence for prion formation. 2005 ed. Proc Natl Acad Sci U S A. 2005;102: 12825–12830. 10.1073/pnas.0506136102 16123127 PMC1200301

[82] Tyedmers J, Mogk A, Bukau B. Cellular strategies for controlling protein aggregation. Nature Rev Mol Cell Biol. 2010;11: 777–788. 10.1038/nrm2993 20944667

[83] Martin A, Baker TA, Sauer RT. Rebuilt AAA + motors reveal operating principles for ATP-fuelled machines. Nature. 2005;437: 1115–1120. 10.1038/nature04031 16237435

[84] Dong J, Castro CE, Boyce MC, Lang MJ, Lindquist S. Optical trapping with high forces reveals unexpected behaviors of prion fibrils. Nature Struct Mol Biol. 2010 ed. 2010;17: 1422–1430. 10.1038/nsmb.1954 21113168 PMC3274366

[85] Kraut DA, Israeli E, Schrader EK, Patil A, Nakai K, Nanavati D, et al Sequence- and species-dependence of proteasomal processivity. ACS Chem Biol. 2012;7: 1444–1453. 10.1021/cb3001155 22716912 PMC3423507

[86] Venkatraman P, Wetzel R, Tanaka M, Nukina N, Goldberg AL. Eukaryotic proteasomes cannot digest polyglutamine sequences and release them during degradation of polyglutamine-containing proteins. Mol Cell. 2004;14: 95–104. 15068806 10.1016/s1097-2765(04)00151-0

[87] Verhoef LGGC, Lindsten K, Masucci MG, Dantuma NP. Aggregate formation inhibits proteasomal degradation of polyglutamine proteins. Hum Mol Genet. 2002;11: 2689–2700. 12374759 10.1093/hmg/11.22.2689

[88] Aza-Blanc P, Ramírez-Weber FA, Laget MP, Schwartz C, Kornberg TB. Proteolysis that is inhibited by hedgehog targets Cubitus interruptus protein to the nucleus and converts it to a repressor. Cell. 1997;89: 1043–1053. 9215627 10.1016/s0092-8674(00)80292-5

[89] Orian A, Schwartz AL, Israël A, Whiteside S, Kahana C, Ciechanover A. Structural motifs involved in ubiquitin-mediated processing of the NF-kappaB precursor p105: roles of the glycine-rich region and a downstream ubiquitination domain. Mol Cell Biol. 1999;19: 3664–3673. 10207090 10.1128/mcb.19.5.3664 PMC84174

[90] Lee C, Schwartz MP, Prakash S, Iwakura M, Matouschek A. ATP-dependent proteases degrade their substrates by processively unraveling them from the degradation signal. Mol Cell. 2001st ed. 2001;7: 627–637. 11463387 10.1016/s1097-2765(01)00209-x

[91] Tian L, RA H, Matouschek A. A conserved processing mechanism regulates the activity of transcription factors Cubitus interruptus and NF-kappaB. Nature Struct Mol Biol. 2005 ed. 2005;12: 1045–1053. 10.1038/nsmb1018 16299518

[92] Hoyt MA, Zich J, Takeuchi J, Zhang M, Govaerts C, Coffino P. Glycine-alanine repeats impair proper substrate unfolding by the proteasome. EMBO J. 2006;25: 1720–1729. 10.1038/sj.emboj.7601058 16601692 PMC1440830

[93] Rape M, Jentsch S. Taking a bite: proteasomal protein processing. Nature Cell Biol. 2002;4: E113–6. 10.1038/ncb0502-e113 11988749

[94] Alexandrov IM, Vishnevskaya AB, Ter-Avanesyan MD, Kushnirov VV. Appearance and propagation of polyglutamine-based amyloids in yeast: tyrosine residues enable polymer fragmentation. J Biol Chem. 2008;283: 15185–15192. 10.1074/jbc.M802071200 18381282 PMC2397454

[95] MacLea KS, Paul KR, Ben-Musa Z, Waechter A, Shattuck JE, Gruca M, et al Distinct amino acid compositional requirements for formation and maintenance of the [PSI⁺] prion in yeast. Mol Cell Biol. 2015;35: 899–911. 10.1128/MCB.01020-14 25547291 PMC4323492

[96] Nakayashiki T, Ebihara K, Bannai H, Nakamura Y. Yeast [PSI+] “prions” that are crosstransmissible and susceptible beyond a species barrier through a quasi-prion state. Mol Cell. 2001;7: 1121–1130. 11430816 10.1016/s1097-2765(01)00259-3

[97] Santoso A, Chien P, Osherovich LZ, Weissman JS. Molecular basis of a yeast prion species barrier. Cell. 2000;100: 277–288. 10660050 10.1016/s0092-8674(00)81565-2

[98] Cherny I, Rockah L, Levy-Nissenbaum O, Gophna U, Ron EZ, Gazit E. The formation of Escherichia coli curli amyloid fibrils is mediated by prion-like peptide repeats. J Mol Biol. 2005;352: 245–252. 10.1016/j.jmb.2005.07.028 16083908

[99] Dueholm MS, Petersen SV, Sønderkær M, Larsen P, Christiansen G, Hein KL, et al Functional amyloid in Pseudomonas. Mol Microbiol. 2010;77: 1009–1020. 10.1111/j.1365-2958.2010.07269.x 20572935

[100] Masel J, Jansen VA, Nowak MA. Quantifying the kinetic parameters of prion replication. Biophys Chem. 1999;77: 139–152. 10326247 10.1016/s0301-4622(99)00016-2

[101] Krauss S, Vorberg I. Prions Ex Vivo: What Cell Culture Models Tell Us about Infectious Proteins. Int J Cell Biol. 2013;2013: 704546–14. 10.1155/2013/704546 24282413 PMC3825132

[102] Zaarur N, Xu X, Lestienne P, Meriin AB, McComb M, Costello CE, et al RuvbL1 and RuvbL2 enhance aggresome formation and disaggregate amyloid fibrils. EMBO J. 2015;34: 2363–2382. 10.15252/embj.201591245 26303906 PMC4570522

[103] Gao X, Carroni M, Nussbaum-Krammer C, Mogk A, Nillegoda NB, Szlachcic A, et al Human Hsp70 Disaggregase Reverses Parkinson's-Linked α-Synuclein Amyloid Fibrils. Mol Cell. 2015;59: 781–793. 10.1016/j.molcel.2015.07.012 26300264 PMC5072489

[104] Chernoff YO, Lindquist SL, Ono B, Inge-Vechtomov SG, Liebman SW. Role of the chaperone protein Hsp104 in propagation of the yeast prion-like factor [*PSI*^*+*^]. Science 1995;268: 880–884. 7754373 10.1126/science.7754373

[105] Storici F, Lewis LK, Resnick MA. In vivo site-directed mutagenesis using oligonucleotides. Nature Biotech. 2001;19: 773–776. 10.1038/90837 11479573

[106] Pezza JA, Langseth SX, Raupp Yamamoto R, Doris SM, Ulin SP, Salomon AR, et al The NatA acetyltransferase couples Sup35 prion complexes to the [PSI+] phenotype. Mol Biol. Cell. 2009;20: 1068–1080. 10.1091/mbc.E08-04-0436 19073888 PMC2633373

