# Supplementary figures and images for: Distinct Prion Domain Sequences Ensure Efficient Amyloid Propagation by Promoting Chaperone Binding or Processing In Vivo
Source: PLoS Genet. 2016 Nov 4;12(11):e1006417. doi: 10.1371/journal.pgen.1006417 (PMC5096688; doi:10.1371/journal.pgen.1006417)

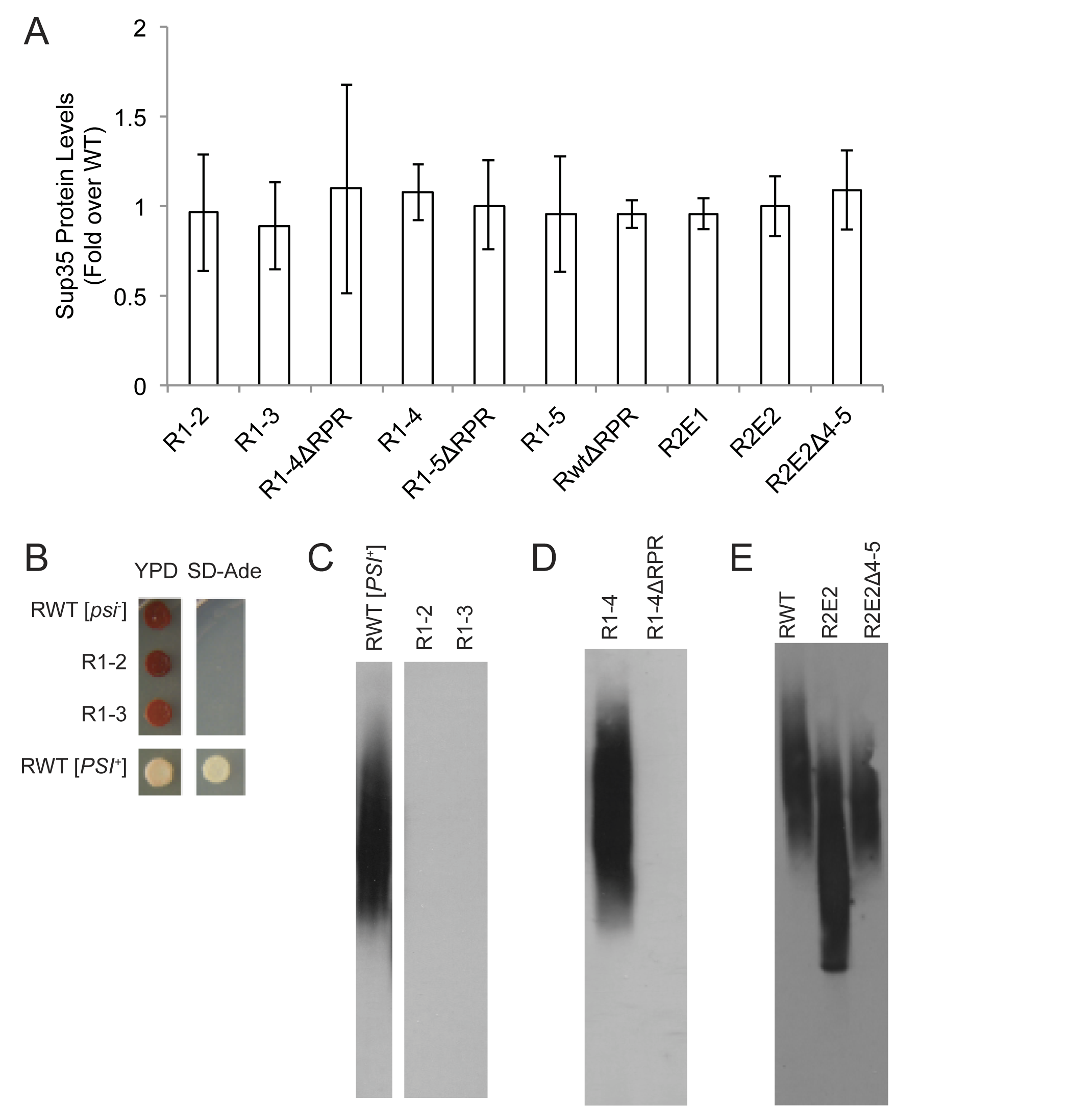

Supplement: S1 Fig — A. Lysates from R1-2 (SY2072), R1-3 (SY2073), R1-4ΔRPR (SY1629), R1-4 (SY2057), R1-5ΔRPR (SY1633), R1-5 (SY2022), ΔRPR (SY2023), R2E1 (SY2247), R2E2 (SY2300), and R2E2Δ4–5 (SY2808) were analyzed by SDS-PAGE and quantitative immunoblotting for Sup35. Bars represent means; error bars represent standard deviations. n≥3. B. R1-2 (SY2072), R1-3 (SY2073) were spotted onto rich medium (YPD) and medium lacking adenine (-Ade) to analyze the [PSI+] phenotype. Wildtype [PSI+] (SLL2606) is shown as a control. C. SDD-AGE was performed on wildtype [PSI+] (SLL2606), R1-2 (SY2072), and R1-3 (SY2073) lysates, followed by immunoblotting for Sup35. Panels shown are non-consecutive lanes run on the same gel. D. SDD-AGE was performed on R1-4 (SY2057) and R1-4ΔRPR (SY1629) lysates, followed by immunoblotting for Sup35. E. SDD-AGE was performed on wildtype [PSI+] (SLL2606), R2E2Δ4–5 (SY2808), and R2E2 (SY2247) lysates, followed by immunoblotting for Sup35. (TIF) [file pgen.1006417.s001.tif]

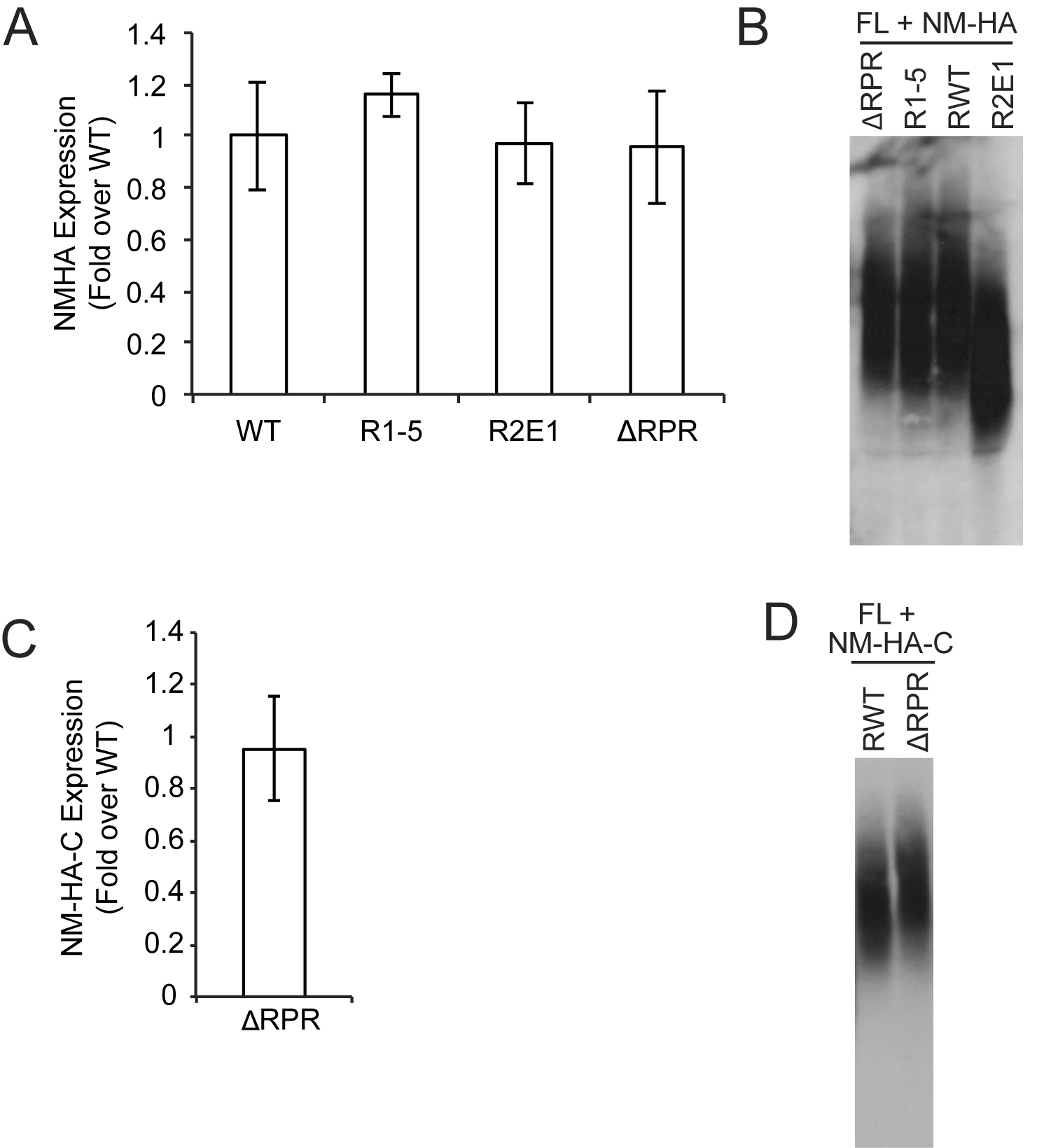

Supplement: S2 Fig — A. Lysates from wildtype (SY3007), R1-5 (SY3008), R2E1 (SY3010), and ΔRPR (SY3009) strains expressing NM-HA were analyzed by SDS-PAGE and quantitative immunoblotting for HA (Bars represent means, error bars represent standard deviations, n≥3). B. SDD-AGE was performed on lysates from wildtype (SY3007), R1-5 (SY3008), R2E1 (SY3010) and ΔRPR (SY3009) strains expressing full-length Sup35 (FL) and NM-HA and immunoblotted for Sup35 n≥3. C. Lysates from wildtype (SY3159), and ΔRPR (SY3164) strains expressing full-length Sup35 (FL) and NM-HA-C were analyzed by SDS-PAGE and quantitative immunoblotting for HA (Bars represent means, error bars represent standard deviations, n≥3). D. SDD-AGE was performed on cell lysates from wildtype (SY3159) and ΔRPR (SY3164), strains expressing full-length Sup35 (FL) and NM-HA-C followed by immunoblotting for Sup35. (TIF) [file pgen.1006417.s002.tif]

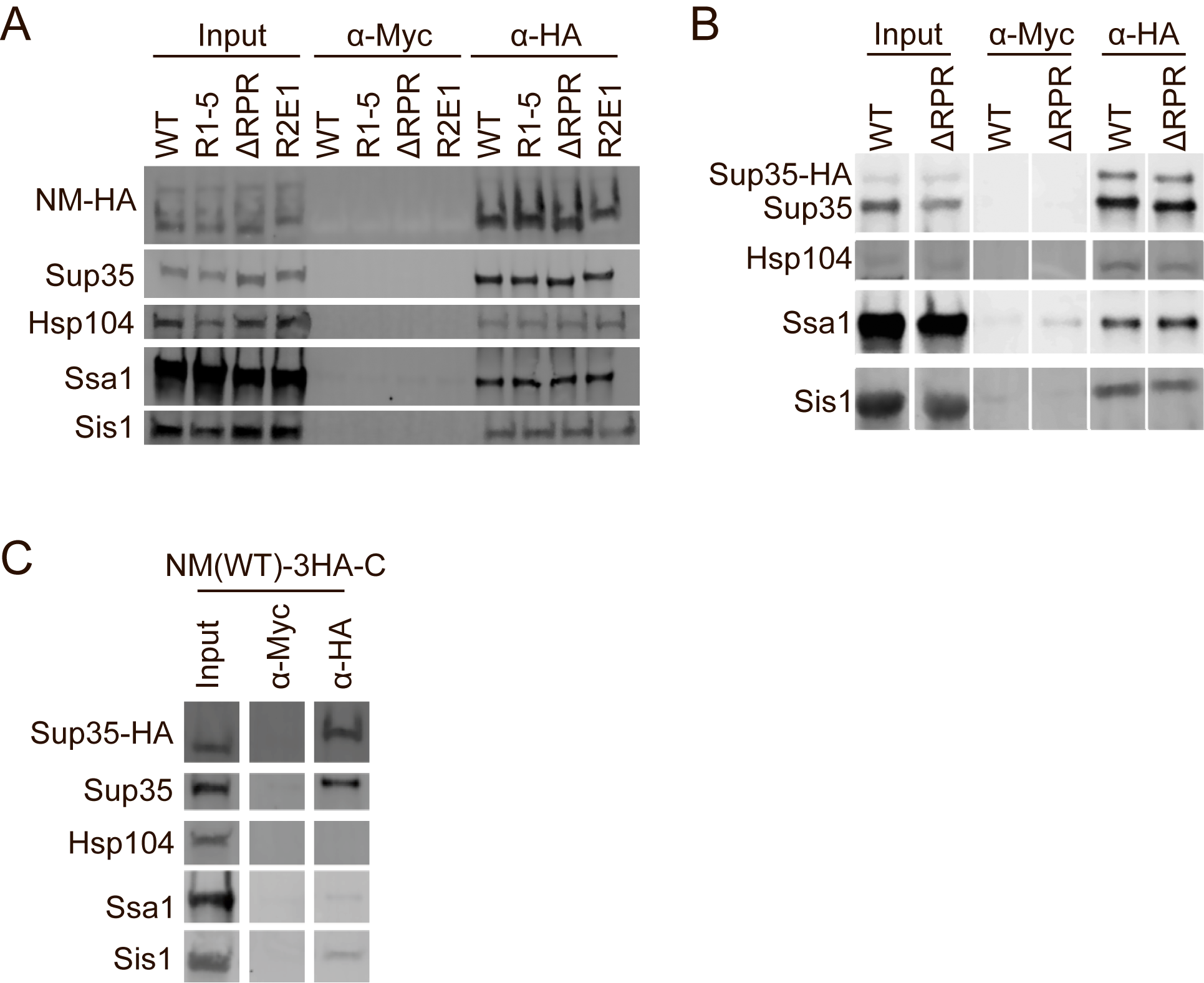

Supplement: S3 Fig — A. Representative gel from experiment quantified in Fig 7A. Aggregates in wildtype (SY3007), R1-5 (SY3008), R2E1 (SY3010), and ΔRPR (SY3009) strains expressing NM-HA and full length Sup35 were immunocaptured using anti-HA magnetic beads and separated by SDS-PAGE and the amount of bound chaperone proteins was determined by immunoblotting. B. Representative gel from experiment quantified in Fig 7B. Aggregates in wildtype (SY3159) and ΔRPR (SY3164) strains expressing NM-HA-C and full length (untagged) Sup35 were immunocaptured with anti-HA magnetic beads and separated by SDS-PAGE, and the amount of bound Hsp104, Ssa1, and Sis1 was determined by immunoblotting. Panels shown are non-consecutive lanes run on the same gel. C. Wildtype (SY3159) strains expressing NM-HA-C and full-length (untagged) Sup35 were treated as in A, and the amount of Hsp104, Ssa1, and Sis1 bound was determined by immunoblotting. Panels shown are non-consecutive lanes run on the same gel. (TIF) [file pgen.1006417.s003.tif]

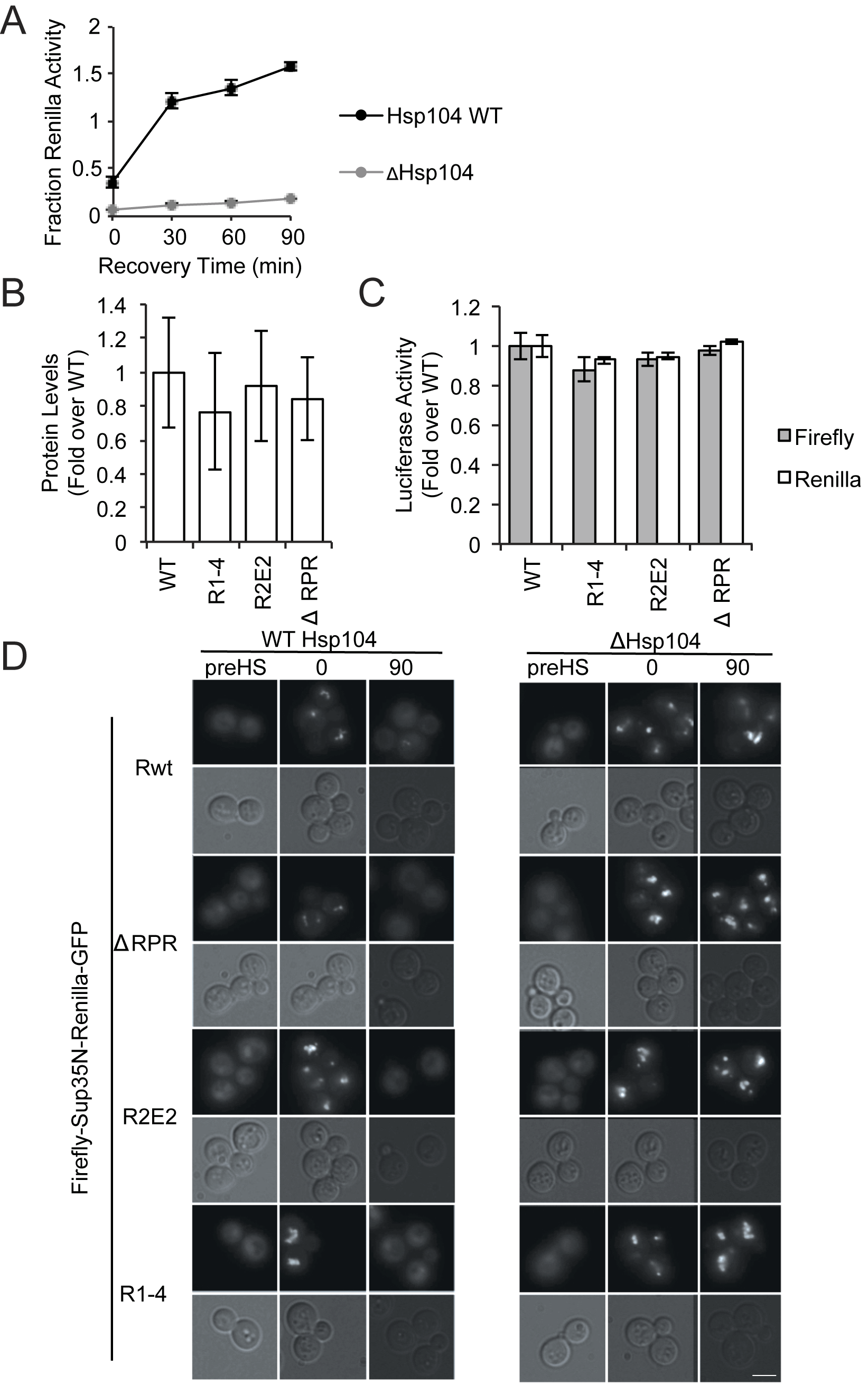

Supplement: S4 Fig — A. Strains containing Renilla luciferase-GFP with or without an Hsp104 disruption (SY2694 and SY2703, respectively) were grown in rich medium, heat shocked at 37°C for 30 min followed by 40°C for 35 minutes with cycloheximide added for the last 10 minutes, and allowed to recover at 30°C in the presence of cycloheximide. Renilla luciferase activity was measured at the indicated time points. n = 3, data represent means; error bars indicate standard deviations. B. Lysates from strains containing Firefly-Sup35N-Renilla-GFP reporters were analyzed by SDS-PAGE and quantitative immunoblotting for GFP. n = 3, bars represent means; error bars represent standard deviations. C. The levels of firefly and Renilla luciferase activity were determined in strains expressing the indicated reporters. n = 3, bars represent means; error bars represent standard deviations. D. Strains containing firefly luciferase-Sup35N(RV)-Renilla luciferase-GFP with or without an Hsp104 disruption were treated as in A, and imaged at the indicated time points. Representative images are shown, scale bar = 5μm. (TIF) [file pgen.1006417.s004.tif]

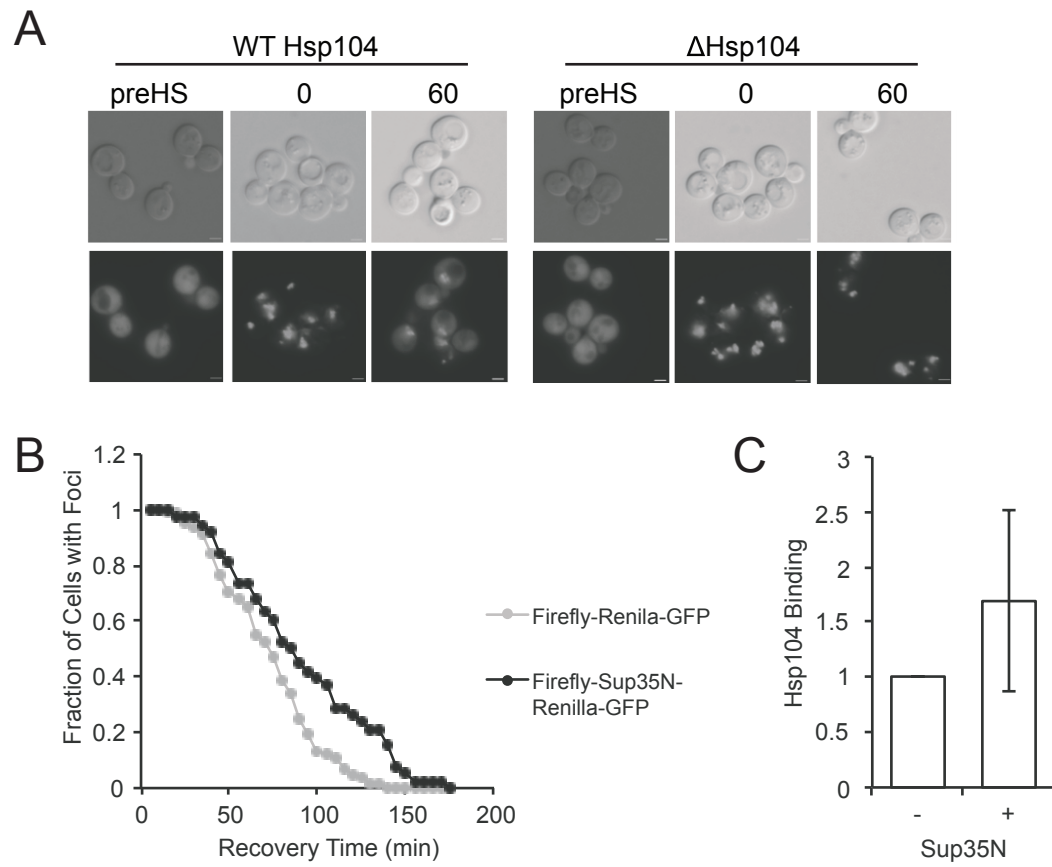

Figure S5

Supplement: S5 Fig — Insertion of Sup35N in dual luciferase reporter delays substrate processing A. A [psi-] strain containing firefly luciferase-Renilla luciferase-GFP with (SY2620) or without (SY2603) an Hsp104 disruption were grown in rich medium, heat shocked at 37°C for 30 min followed by 40°C for 35 minutes with cycloheximide added for the last 10 minutes, and allowed to recover at 30°C in the presence of cycloheximide, and imaged at the indicated time points. Representative images are shown. Scale bar = 2μm. B. The indicated strains expressing the dual luciferase reporter without (SY2597) and with (SY2603) Sup35N were incubated at 37°C for 30 minutes, followed by 40°C for 35 minutes, with cycloheximide added for the last 10 minutes, in a microfluidics chamber. Cells were then allowed to recover at 30°C in the presence of cycloheximide, and images were taken at the indicated time points. At each time point, the percentage of cells containing GFP foci was determined. n≥38. C. Dual luciferase reporter fusion proteins without (SY2597) and with (SY2603) Sup35N were immunocaptured, separated by SDS-PAGE and the amount of bound Hsp104 was quantified by western blot. n = 6, bars represent means; error bars represent standard deviations. (PDF) [file pgen.1006417.s005.pdf]
