# Supplementary material for: Distinct Prion Domain Sequences Ensure Efficient Amyloid Propagation by Promoting Chaperone Binding or Processing In Vivo
Source: PLoS Genet. 2016 Nov 4;12(11):e1006417. doi: 10.1371/journal.pgen.1006417 (PMC5096688; doi:10.1371/journal.pgen.1006417)
Supplement: S1 Text — (DOCX) [file pgen.1006417.s006.docx]

**Supplementary Materials**

**Nucleated Polymerization Model:**

The dynamics of prion aggregates are typically modeled by the nucleated polymerization model (NPM) first introduced by Masel, Jensen and Nowak (1999) for the PrP prion in mammals and later adapted for use in the study of the yeast prion [*PSI*^+^] (Derdowski et al, 2010; Davis & Sindi, 2015). In this model, normal protein is converted to the prion form through contact with existing aggregates. Existing aggregates may also fragment into two smaller aggregates, soluble protein is continually created and both normal and aggregated protein can be diluted through the cell division process.

The dynamics of protein under the NPM are typically modeled by an infinite system of ordinary differential equations tracking time-evolution of the concentration of normal protein, $x(t)$, and the concentration of aggregates of every possible size $i$, $y_{i}(t)$, as follows:

$$\frac{dx}{dt}=\alpha-\mu x\left( t \right)-2\beta x\left( t \right)\sum_{i=n_{0}}^{\infty} y_{i}\left( t \right)+\gamma n_{0}\left( n_{0}-1 \right)\sum_{i=n_{0}}^{\infty} y_{i}\left( t \right)$$

$$\frac{dy_{i}}{dt}=-{\mu y}_{i}\left( t \right)-2\beta\left( y_{i}\left( t \right)-y_{i-1}\left( t \right) \right)-\gamma\left( i-1 \right)y_{i}\left( t \right)+2\gamma\sum_{j=\left( i+1 \right)}^{\infty} y_{j}\left( t \right).$$

In these equations $\alpha$ is the constant rate of synthesis of normal protein; $\beta,$ the rate that aggregates convert normal protein; $\mu$, the rate of dilution through cellular division and $\gamma$, the rate of fragmentation. We note that aggregates are considered to be linear fibers and fragmentation is modeled to occur between any adjacent monomers. Finally, in the NPM we assume that there is a minimal stable aggregate size of $n_{0}$. That is, if an aggregate is fragmented below this minimal size, the protein monomers will return to the soluble pool.

When the size-distribution is not explicitly needed, the NPM is often analyzed by considering the total number of aggregates, $Y\left( t \right)= \sum_{i=n_{0}}^{\infty} y_{i}(t)$, and the total concentration of aggregated protein, $Z\left( t \right)=\sum_{i=n_{0}}^{\infty} i y_{i}(t)$. Under these conditions, the NPM reduces from an infinite set of ODEs to the following set of three ODEs:

$$\frac{dx}{dt}=\alpha-\mu x\left( t \right)-2\beta x\left( t \right)Y\left( t \right)+\gamma n_{0}\left( n_{0}-1 \right)Y\left( t \right)$$

$$\frac{dY}{dt}=-\left( \mu+\gamma\left( 2n_{0}-1 \right) \right)Y\left( t \right)+\gamma Z\left( t \right)$$

$$\frac{dZ}{dt}=-\mu Z\left( t \right)+2\beta x\left( t \right)Y\left( t \right)- \gamma n_{0}\left( n_{0}-1 \right)Y\left( t \right).$$

This is reduction is also called the “moment-closure” because the aggregate distribution is replaced by the zeroth ($Y\left( t \right)$) and first ($Z\left( t \right)$) moments of the aggregate size distribution.

**R_0_ Value of Prion Aggregates:**

Using a PDE version of the NPM, Prüss et al (2006) observed that the reduced system $\left( x\left( t \right),Y\left( t \right),Z(t) \right)$ could be understood as a standard epidemiological model. In this case the system behavior can be understood by the basic reproductive number $R_{0}$. This value arises in many epidemic models and is the number of secondary infections created by a single infectious entity during its lifetime. When $R_{0}>1$, a disease will persist stably and when $R_{0}<1$ the disease will die out.

In particular, when the number of aggregates is small and the pool of soluble protein large, as we will detail below, the rate of aggregate amplification will be a simple function of $R_{0}$. Finally, we note an interplay between the $R_{0}$ and the steady-state fraction of normal protein, $x^{*}$, in the system

$$x^{*}=\frac{\mu\left( \mu+\gamma(n_{0}-1) \right)\left( \mu+\gamma n_{0} \right)}{2\alpha\beta\gamma},$$

namely, $R_{0}=1/x^{*}$ (Davis & Sindi, 2015). This behavior between the fraction of normal (i.e. healthy) protein remaining at steady-state and the $R_{0}$ has been previously reported for the classic SIR epidemic models (Brauer & Castillo-Chavez, 2001).

Because the prion strains under consideration do not differ in the rate of dilution, synthesis, minimum stable aggregate size or conversion, we are able to interpret differences in the steady-state fraction of soluble protein and the rate of aggregate amplification (that we understand to be related to the above $R_{0}$ value) to be due to differences in the rate of fragmentation alone.

**Rate of Aggregate Amplification:**

To demonstrate the relationship between the $R_{0}$ value and the initial rate of aggregate amplification, we will analyze the behavior of the system very close to the disease-free steady state. For simplicity, we assume that the total concentration of Sup35 is at its steady state value of $\frac{\alpha}{\mu}$. This means throughout all time we have:

$$x\left( t \right)+Z\left( t \right)=\frac{\alpha}{\mu}.$$

This conservation will allow us to remove the variable $Z\left( t \right)$, since it can always be determined from $x\left( t \right)$. As such our system of 3 ODEs is equivalent to the following system:

$$\frac{dx}{dt}=\alpha-\mu x\left( t \right)-2\beta x\left( t \right)Y\left( t \right)+ \gamma n_{0}\left( n_{0}-1 \right)Y\left( t \right)$$

$$\frac{dY}{dt}=-\left( \mu+\gamma\left( 2n_{0}-1 \right) \right)Y\left( t \right)+\gamma\left( \frac{\alpha}{\mu}-x\left( t \right) \right).$$

The rate of amplification of aggregates upon the introduction of a small amount of prion/aggregated protein is governed by the eigenvalues of the Jacobian of the system evaluated at the disease-free steady-state $\left( x\left( 0 \right),Y\left( 0 \right) \right)=(\frac{\alpha}{\mu},0)$:

$J\left( x,Y \right)= \left[ \begin{matrix} -\mu-2\beta Y & -2\beta x+ \gamma n_{0}\left( n_{0}-1 \right) \\ -\gamma& -\left( \mu+\gamma\left( 2n_{0}-1 \right) \right) \end{matrix} \right]$.

When we evaluate the Jacobian at the point$\left( x\left( 0 \right),Y\left( 0 \right) \right)=(\frac{\alpha}{\mu},0)$ we have the matrix:

$J\left( \frac{\alpha}{\mu},0 \right)= \left[ \begin{matrix} -\mu& -\frac{2\beta\alpha}{\mu}+ \gamma n_{0}\left( n_{0}-1 \right) \\ -\gamma& -\left( \mu+\gamma\left( 2n_{0}-1 \right) \right) \end{matrix} \right]$.

The eigenvalues of this matrix are solutions to the characteristic equation and are given by

$$\lambda_{1,2}=\frac{1}{2}\left( \gamma-2n0\gamma-2\mu\pm\frac{\sqrt{\gamma}\sqrt{8\alpha\beta+\gamma\mu}}{\sqrt{\mu}} \right).$$

We note that the condition requiring that one of the eigenvalues is positive, and thus that there is an attracting steady-state where prion aggregate persist, is equivalent to the condition that $R_{0}>1.$ Further, this positive eigenvalue is precisely the rate of aggregate amplification. We note that this eigenvalue can be written as a function of $R_{0}:$

$$\lambda=\sqrt{R_{0}\left( \gamma\mu+\mu^{2} \right)+\frac{\gamma^{2}}{4}}-\left( \gamma n_{0}+\mu\right)+\frac{\gamma}{2}.$$

When the value of $\gamma\ll1$ (i.e., fragmentation rate is small), we have a useful approximation:

$\lambda\approx\left( \sqrt{R_{0}}-1 \right)\mu$.

**Comparison to Previous Analyses:**

Previously, Tanaka et al. (2006) compared the rate of aggregate amplification upon recovery from GdnHCl. However, we note some differences between their observations and this mathematical analysis of the rate of aggregate amplification.

First, Tanaka et al. (2006) did not consider that aggregates smaller than the critical size, $n_{0}$, to re-enter the soluble pool. This could be considered equivalent to assuming that either the fragmentation rate was very small ($\gamma\ll1$) or that aggregates had minimal size $n_{0}=1$. Second, we note that their differential equation model assumed that aggregate fragmentation occurred as a rate proportional to the total aggregated protein, $Z\left( t \right)$, rather than total number of fragmentation sites, $Z\left( t \right)-Y(t)$.

Despite the technical issues with their model, because they considered $\gamma\ll1$ they correctly concluded that simply a function of the product of $\beta\gamma$. (We note that 2$\beta$ in our model is equivalent to their $\beta$ because we explicitly consider each end of an aggregate as a site for conversion.) Because $R_{0}$ itself is specified as follows:

$R_{0}=\frac{2\alpha\beta\gamma}{\mu\left( \mu+\gamma(n_{0}-1) \right)\left( \mu+\gamma n_{0} \right)} \approx\frac{2\alpha\beta\gamma}{\mu^{3}}$,

the rate of aggregate amplification is approximately given by:

$$\lambda\approx\left( \sqrt{R_{0}}-1 \right)\mu\approx\sqrt{\frac{2\alpha\beta\gamma}{\mu}}-\mu.$$

Thus, our more detailed analysis supports the previous observations from Tanaka et. al. (2006). Namely, when fixing the rate of protein synthesis, ($\alpha$), and dilution ($\mu$), the product $\beta\gamma$ determines the aggregate amplification rate.

**Results:** We first note statistically significant differences in the $R_{0}$ values for each of the conditions studied. As mentioned above, we know that the steady-state concentration of Sup35 is the inverse of the $R_{0}$ value. Thus, we have estimates for $R_{0}$ values for each condition (See Figure 1). We compared these values between strains with a 2 sample t-test (Matlab ttest2 function). P-values are given in Table 1 (Soluble Sup35) and Table 2 ($R_{0}$ values) for a significance level below 0.05 and an “NA” is shown when no significance is found.

**References:**

Brauer, F., & Castillo-Chavez, C. (2001). *Mathematical models in population biology and epidemiology* (Vol. 40, pp. xxiv+-416). New York: Springer.

Davis, J. K., & Sindi, S. S. (2015). A mathematical model of the dynamics of prion aggregates with chaperone-mediated fragmentation. *Journal of mathematical biology*, 1-24.

Derdowski, A., Sindi, S. S., Klaips, C. L., DiSalvo, S., & Serio, T. R. (2010). A size threshold limits prion transmission and establishes phenotypic diversity. *Science*, *330*(6004), 680-683.

Masel, J., Jansen, V. A., & Nowak, M. A. (1999). Quantifying the kinetic parameters of prion replication. *Biophysical chemistry*, *77*(2), 139-152.

Prüss, J., Pujo-Menjouet, L., Webb, G., & Zacher, R. (2006). Analysis of a model for the dynamics of prions. *Discrete Contin. Dyn. Syst. Ser. B*, *6*(1), 225-235.

Sindi, S.S. (In Preparation). A Logistic Model of Yeast Prion Aggregate Dynamics to Facilitate Estimation of Kinetic Parameters from Experimental Data.

Tanaka, M., Collins, S. R., Toyama, B. H., & Weissman, J. S. (2006). The physical basis of how prion conformations determine strain phenotypes. *Nature*, *442*(7102), 585-589.
