# Supplementary material for: Distinct Prion Domain Sequences Ensure Efficient Amyloid Propagation by Promoting Chaperone Binding or Processing In Vivo
Source: PLoS Genet. 2016 Nov 4;12(11):e1006417. doi: 10.1371/journal.pgen.1006417 (PMC5096688; doi:10.1371/journal.pgen.1006417)
Supplement: S1 Table — (DOCX) [file pgen.1006417.s007.docx]

**S1 Table. Significantly Different Levels of Soluble Sup35 (%)**

|  | **R15ΔRPR** | **R1-5** | **WTΔRPR** | **WT** | **R2E1** | **R2E2** |
| --- | --- | --- | --- | --- | --- | --- |
| **R15ΔRPR** | - | - | - | - | - | - |
| **R1-5** | 1.28 x 10^-4^ | - | - | - | - | - |
| **WTΔRPR** | 0.0323 | 0.0024 | - | - | - | - |
| **WT** | 1.71 x 10^-4^ | NA | 0.0101 | - | - | - |
| **R2E1** | 7.19 x 10^-4^ | 0.0401 | 0.0021 | NA | - | - |
| **R2E2** | 0.0012 | 0.0029 | 0.0018 | 0.0326 | NA | - |
