# Supplementary material for: Distinct Prion Domain Sequences Ensure Efficient Amyloid Propagation by Promoting Chaperone Binding or Processing In Vivo
Source: PLoS Genet. 2016 Nov 4;12(11):e1006417. doi: 10.1371/journal.pgen.1006417 (PMC5096688; doi:10.1371/journal.pgen.1006417)
Supplement: S2 Table — (DOCX) [file pgen.1006417.s008.docx]

**S2 Table. Significantly Different R_0_ Values**

|  | **R15ΔRPR** | **R1-5** | **WTΔRPR** | **WT** | **R2E1** | **R2E2** |
| --- | --- | --- | --- | --- | --- | --- |
| **R15ΔRPR** | - | - | - | - | - | - |
| **R1-5** | 9.33 x 10^-4^ | - | - | - | - | - |
| **WTΔRPR** | 0.0323 | 0.0077 | - | - | - | - |
| **WT** | 0.0081 | NA | 0.0276 | - | - | - |
| **R2E1** | 0.0115 | 0.0354 | 0.0175 | NA | - | - |
| **R2E2** | 0.0025 | 0.0021 | 0.0033 | 0.0023 | NA | - |
