# Supplementary material for: Distinct Prion Domain Sequences Ensure Efficient Amyloid Propagation by Promoting Chaperone Binding or Processing In Vivo
Source: PLoS Genet. 2016 Nov 4;12(11):e1006417. doi: 10.1371/journal.pgen.1006417 (PMC5096688; doi:10.1371/journal.pgen.1006417)
Supplement: S3 Table — (DOCX) [file pgen.1006417.s009.docx]

**S3 Table. Plasmids**

| **Name** | **Description** | **Reference** |
| --- | --- | --- |
| **SLL6682** | pRS306-P_SUP35_Sup35C | Zhou *et al.* 2001 |
| **SLL6686** | pRS306-P_SUP35_Sup35 | DiSalvo *et al.* 2011 |
| **SB237** | pRS305-P_GPD_ | This study |
| **SB531** | pRS304-P_GPD_GST(UGA)DsRedNLS | Pezza, et. al., 2009 |
| **SB526** | pFA6a-KanMX6-P_MFA1_ | Pezza, et. al., 2009 |
| **SB537** | pRS426-P_SUP35_NM-3HA-C | DePace, et. al., 1998 |
| **SB549** | pRS306-P_SUP35_Sup35R1-4ΔRPR | This study |
| **SB550** | pRS306-P_SUP35_Sup35R1-5ΔRPR | This study |
| **SB653** | pRS305-P_SUP35_NM-3HA | Pezza, et. al., 2015 |
| **SB775** | pRS306-P_SUP35_Sup35R1-4 | This study |
| **SB776** | pRS306-P_SUP35_Sup35R1-5 | This study |
| **SB777** | pRS306-P_SUP35_Sup35ΔRPR | This study |
| **SB787** | pRS306-P_SUP35_Sup35R2E1 | This study |
| **SB803** | pRS306-P_SUP35_Sup35R1-2 | This study |
| **SB804** | pRS306-P_SUP35_Sup35R1-3 | This study |
| **SB859** | pRS306-P_SUP35_Sup35R2E2 | This study |
| **SB883** | pRS303-P_SUP35_Sup35R2E1 | This study |
| **SB884** | pRS303-P_SUP35_Sup35R2E2 | This study |
| **SB910** | pRS304-P_GPD_GST(UGA)YFPNLS | This study |
| **SB973** | pRS306-P_GPD_Firefly-Renilla-GFP | This study |
| **SB975** | pRS306-P_GPD_Firefly-Sup35NΔRPR-Renilla-GFP | This study |
| **SB976** | pRS306-P_GPD_Firefly-Sup35NR2E2-Renilla-GFP | This study |
| **SB985** | pRS306-P_GPD_Firefly-Sup35NR1-4-Renilla-GFP | This study |
| **SB986** | pRS306-P_GPD_Renilla-GFP | This study |
| **SB994** | pRS306-P_GPD_Firefly-Sup35N-Renilla-GFP | This study |
| **SB1008** | pRS306-P_SUP35_Sup35R2E2Δ4-5 | This study |
| **SB1040** | pRS305-P_SUP35_NM(R1-5)-3HA | This study |
| **SB1041** | pRS305-P_SUP35_NM(ΔRPR)-3HA | This study |
| **SB1042** | pRS305-P_SUP35_NM(R2E1)-3HA | This study |
| **SB1090** | pRS305- P_SUP35_NM-3HA-C | This study |
| **SB1092** | pRS305- P_SUP35_NM(ΔRPR)-3HA-C | This study |
