# Supplementary material for: Distinct Prion Domain Sequences Ensure Efficient Amyloid Propagation by Promoting Chaperone Binding or Processing In Vivo
Source: PLoS Genet. 2016 Nov 4;12(11):e1006417. doi: 10.1371/journal.pgen.1006417 (PMC5096688; doi:10.1371/journal.pgen.1006417)
Supplement: S4 Table — (DOCX) [file pgen.1006417.s010.docx]

**S4 Table. Oligonucleotide sequences**

| **Name** | **Sequence** |
| --- | --- |
| **5BamHISup35** | 5’- GGATCCATGTCGGATTCAAACCAAGGC-3’ |
| **3R1-2EcoRV** | 5’-GATATCTTGCAAATTGTTATTGTAGTTGAAGTTTTTGTA  ATTTCCACGATTGTACTGTT-3’ |
| **3R1-3EcoRV** | 5’- GATATCTTGCAAATTGTTATTGTAGTTGAAGTTTTTGTA  ATTTCCACGATTATACTGTT-3’ |
| **3R1-4EcoRV** | 5’- GATATCTTGCAAATTGTTATTGTAGTTGAAGTTTTTGTA  ATTTCCACGATTGTACTGTT-3’ |
| **3R1-5EcoRV** | 5’-GATATCTTGCAAATTGTTATTGTAGTTGAAGTTTTTGTA  ATTTCCACGATTGAATTGCT-3’ |
| **3Sup35R1-6EcoRV** | 5’- GATATCCGCCACCTTGTGGATTGAATTC-3’ |
| **R2E1 insertF** | 5’- GTGGCTATCAACAGTACAACCAACAAG-3’ |
| **R2E1 insertR** | 5’- TTGGTTGTACTGTTGATAGCCACCTTG-3’ |
| **R2BstXI QCF** | 5’-CAAAATTACCAAGGTTATTCTGGGTATCAACAAGGTGG  CTATCAACAGTAC-3’ |
| **R2BstXI QCR** | 5’- GTACTGTTGATAGCCACCTTGTTGATACCCAGAATAAC  CTTGGTAATTTTG-3’ |
| **5EcoRI Citrine** | 5’-GATATCATGTCTAAAGGTGAAGAATTATTC-3’ |
| **3ClaI CitrineNLS** | 5’-ATCGATTTATCCCTTTGGGTCTTCAACCTTTCTCTTCT  TCTTTGGTGGGGTAGAGTGCCCTTTGTACAATTCATCCATACCATG-3’ |
| **5XbaI firefly** | 5’-TCTAGAATGGAAGATGCCAAAAACATTAAG-3’ |
| **3BamHI Firefly** | 5’-GGATCCACCTTGAGACTGTGGTTGGAAAC-3’ |
| **5BamHIGs3 Sup35N** | 5’-GGATCCGGTAGTGGTAGTGGTAGTATGTCGGATTCAAA  CCAAGGC-3’ |
| **3BamHISup35N** | 5’-GGATCCACCTTGAGACTGTGGTTGG-3’ |
| **5BamHIGS3 Renilla** | 5’-GGATCCGGTAGTGGTAGTGGTAGTATGACTTCGAAAGTT  TATGATCC-3’ |
| **3EcoRI Renilla** | 5’-GAATTCTTGTTCATTTTTGAGAACTC-3’ |
| **5EcoRI GS3 GFP** | 5’-GAATTCGGTAGTGGTAGTGGTAGTATGGCTAGCAAAGG  AGAA-3’ |
| **3XhoIGArGFP** | 5’-CTCGAGTTAACCTGCACCTGCACCACCACCTGCACCTGC  TTTGTATAGTTCATCCATGCC-3’ |
| **5XbaIRenilla** | 5’-TCTAGAATGACTTCGAAAGTTTATGATCC-3’ |
| **5Sup35Nrepck** | 5’-TGTCGGATTCAAACCAAGGCAACAATCAGCAAAAC-3’ |
| **3Sup35Nrepck** | 5’-GCCAACCTTCTTGGTAGCATTGGCCAACTTGATACC-3’ |
| **F4-Psup35** | 5’-CTTCTCTTGAAAGACTCCATTGTACTGTAACAAAAAGCGG  GAATTCGAGCTCGTTTAAAC-3’ |
| **R2-PMFA1** | 5’-GCTGGTAGTTTTGCTGATTGTTGCCTTGGTTTGAATCCGA  CATGGATCCTTCTATTGAT-3’ |
