# Supplementary material for: Distinct Prion Domain Sequences Ensure Efficient Amyloid Propagation by Promoting Chaperone Binding or Processing In Vivo
Source: PLoS Genet. 2016 Nov 4;12(11):e1006417. doi: 10.1371/journal.pgen.1006417 (PMC5096688; doi:10.1371/journal.pgen.1006417)
Supplement: S5 Table — (DOCX) [file pgen.1006417.s011.docx]

**S5 Table. Strains**

| **Strain** | **Genotype** | **Plasmids Integrated** | **Reference** |
| --- | --- | --- | --- |
| **SLL2606** | *MATa* [*PSI*^+^] *ade1-14 his3Δ200 trp1-289 ura3-52 leu2-3, 112* | - | Chernoff et al. 1995 |
| **SLL2119** | *MATa* [*psi*^–^] *ade1-14 his3Δ200 trp1-289 ura3-52 leu2-3, 112* | - | Chernoff et al. 1995 |
| **SLL3250** | *MATα* [*PSI*^+^] *ade1-14 his3Δ200 trp1-289 ura3-52 leu2-3, 112* | - | Chernoff et al. 1995 |
| **SY1629** | *MATa* [*psi*^–^] *ade1-14 his3Δ200 trp1-289 ura3-52 leu2-3, 112 SUP35R1-4ΔRPR* | SB549 | This study |
| **SY1633** | *MATa* [*PSI*^+^] *ade1-14 his3Δ200 trp1-289 ura3-52 leu2-3, 112 SUP35R1-5 ΔRPR* | SB550 | This study |
| **SY2022** | *MATa* [*PSI*^+^] *ade1-14 his3Δ200 trp1-289 ura3-52 leu2-3, 112 SUP35R1-5* | SB776 | This study |
| **SY2023** | *MATa* [*PSI*^+^] *ade1-14 his3Δ200 trp1-289 ura3-52 leu2-3, 112 SUP35ΔRPR* | SB777­ | This study |
| **SY2057** | *MATa* [*PSI*^+^] *ade1-14 his3Δ200 trp1-289 ura3-52 leu2-3, 112 SUP35R1-4* | SB775 | This study |
| **SY2072** | *MATa* [*psi*^–^] *ade1-14 his3Δ200 trp1-289 ura3-52 leu2-3, 112 SUP35R1-2* | SB803 | This study |
| **SY2073** | *MATa* [*psi*^–^] *ade1-14 his3Δ200 trp1-289 ura3-52 leu2-3, 112 SUP35R1-3* | SB804 | This study |
| **SY2212** | *MATa* [*psi*^–^] *ade1-14 his3Δ200 trp1-289 ura3-52 leu2-3, 112 SUP35 ΔRPR* | SB777 | This study |
| **SY2215** | *MATa* [*psi*^–^] *ade1-14 his3Δ200 trp1-289 ura3-52 leu2-3, 112 SUP35R1-5* | SB776 | This study |
| **SY2247** | *MATa* [*PSI*^+^] *ade1-14 his3Δ200:: HIS3::P_SUP35_SUP35R2E1 trp1-289 ura3-52 leu2-3, 112 SUP35R2E1* | SB787, SB883 | This study |
| **SY2300** | *MATa* [*PSI*^+^] *ade1-14 his3Δ200:: HIS3::P_SUP35_SUP35R2E2 trp1-289 ura3-52 leu2-3, 112 sup35::P_MFA1_SUP35R2E2::KANMX6* | SB859, SB884 | This study |
| **SY2302** | *MATa* [*psi*^–^] *ade1-14 his3Δ200::HIS3:: P_SUP35_SUP35R2E1 trp1-289 ura3-52 leu2-3, 112 SUP35R2E1* | SB787, SB883 | This study |
| **SY2393** | *MATα* [*psi*^–^] *ade1-14 his3Δ200 trp1-289:: TRP1:: P_GPD_GST(UGA)YFPNLS ura3-52 leu2-3, 112* | SB910 | This study |
| **SY2461** | *MATα* [*psi*^–^] *ade1-14 his3Δ200 trp1-289:: TRP1:: P_GPD_GST(UGA)YFPNLS ura3-52 leu2-3, 112 SUP35R1-5* | SB776, SB910 | This study |
| **SY2463** | *MATα* [*psi*^–^] *ade1-14 his3Δ200 trp1-289:: TRP1:: P_GPD_GST(UGA)YFPNLS ura3-52 leu2-3, 112 SUP35ΔRPR* | SB777, SB910 | This study |
| **SY2465** | *MATα* [*psi*^–^] *ade1-14 his3Δ200:: HIS3::P_SUP35_SUP35R2E1 trp1-289:: TRP:: P_GPD_GST(UGA)YFPNLS ura3-52 leu2-3, 112 SUP35R2E1* | SB787, SB910 | This study |
| **SY2466** | *MATa* [*PSI*^+^] *ade1-14 his3Δ200 trp1-289 ura3-52::URA3::P_SUP35_Sup35C leu2-3, 112* | SLL6682 | This study |
| **SY2467** | *MATa* [*PSI*^+^] *ade1-14 his3Δ200 trp1-289 ura3-52::URA3::P_SUP35_Sup35C leu2-3, 112 SUP35R1-4* | SLL6682 | This study |
| **SY2597** | *MATa* [*psi*^–^] *ade1-14 his3Δ200 trp1-289 ura3-52::URA3::P_GPD_Firefly-Renilla-GFP leu2-3, 112* | SB973 | This study |
| **SY2603** | *MATa* [*psi*^–^] *ade1-14 his3Δ200 trp1-289 ura3-52::URA3::P_GPD_Firefly-SUP35N-Renilla-GFP leu2-3, 112* | SB994 | This study |
| **SY2620** | *MATa* [*psi*^–^] *ade1-14 his3Δ200 trp1-289 ura3-52::URA3::P_GPD_Firefly-Renilla-GFP leu2-3, 112 hsp104::LEU2* | SB973 | This study |
| **SY2625** | *MATa* [*psi*^–^] *ade1-14 his3Δ200 trp1-289 ura3-52::URA3::P_GPD_Firefly-SUP35N-Renilla-GFP leu2-3, 112 hsp104::LEU2* | SB994 | This study |
| **SY2637** | *MATa* [*psi*^–^] *ade1-14 his3Δ200 trp1-289 ura3-52::URA::P_GPD_Firefly-SUP35NΔRPR-Renilla-GFP leu2-3, 112* | SB975 | This study |
| **SY2640** | *MATa* [*psi*^–^] *ade1-14 his3Δ200 trp1-289 ura3-52::URA3::P_GPD_Firefly-SUP35NR2E2-Renilla-GFP leu2-3, 112* | SB976 | This study |
| **SY2651** | *MATa* [*psi*^–^] *ade1-14 his3Δ200 trp1-289 ura3-52::URA3::P_GPD_Firefly-SUP35NΔRPR-Renilla-GFP leu2-3, 112 hsp104::LEU2* | SB975 | This study |
| **SY2654** | *MATa* [*psi*^–^] *ade1-14 his3Δ200 trp1-289 ura3-52::URA3::P_GPD_Firefly-SUP35NR2E2-Renilla-GFP leu2-3, 112 hsp104::LEU2* | SB976 | This study |
| **SY2666** | *MATa* [*psi*^–^] *ade1-14 his3Δ200 trp1-289 ura3-52::URA3::P_GPD_Firefly-SUP35NR1-4-Renilla-GFP leu2-3, 112* | SB985 | This study |
| **SY2679** | *MATa* [*psi*^–^] *ade1-14 his3Δ200 trp1-289 ura3-52::URA3::P_GPD_Firefly-SUP35NR1-4-Renilla-GFP leu2-3, 112 hsp104::LEU2* | SB985 | This study |
| **SY2694** | *MATa* [*psi*^–^] *ade1-14 his3Δ200 trp1-289 ura3-52::URA3::P_GPD_Renilla-GFP leu2-3, 112* | SB986 | This study |
| **SY2703** | *MATa* [*psi*^–^] *ade1-14 his3Δ200 trp1-289 ura3-52::URA3::P_GPD_Renilla-GFP leu2-3, 112 hsp104::LEU2* | SB986 | This study |
| **SY2808** | *MATa* [*PSI*^+^] *ade1-14 his3Δ200 trp1-289 ura3-52 leu2-3, 112 SUP35R2E2Δ4-5* | SB1008 | This study |
| **SY2991** | *MATα* [*psi*^–^] *ade1-14 his3Δ200 trp1-289 ura3-52 leu2-3, 112 HSP104Y662F* | - | This study |
| **SY3001** | *MATa* [*PSI*^+^] *ade1-14 his3Δ200 trp1-289 ura3-52 leu2-3, 112 HSP104Y662F* | - | This study |
| **SY3002** | *MATa* [*PSI*^+^] *ade1-14 his3Δ200 trp1-289 ura3-52 leu2-3, 112 SUP35R1-5 HSP104Y662F* | SB776 | This study |
| **SY3004** | *MATa* [*PSI*^+^] *ade1-14 his3Δ200 trp1-289 ura3-52 leu2-3, 112 SUP35ΔRPR HSP104Y662F* | SB777 | This study |
| **SY3005** | *MATa* [*PSI*^+^] *ade1-14 his3Δ200 trp1-289 ura3-52 leu2-3, 112 SUP35R2E1 HSP104Y662F* | SB787 | This study |
| **SY3007** | *MATa* [*PSI*^+^] *ade1-14 his3Δ200 trp1-289 ura3-52, leu2-3, 112::LEU2::P_SUP35_NM-3HA* | SB653 | This study |
| **SY3008** | *MATa* [*PSI*^+^] *ade1-14 his3Δ200 trp1-289 ura3-52 leu2-3, 112::LEU2:: ::P_SUP35_NM(R1-5)-3HA SUP35R1-5* | SB776, SB1040 | This study |
| **SY3009** | *MATa* [*PSI*^+^] *ade1-14 his3Δ200 trp1-289 ura3-52 leu2-3, 112::LEU2:: ::P_SUP35_NM(ΔRPR)-3HA SUP35ΔRPR* | SB777, SB1041 | This study |
| **SY3010** | *MATa* [*PSI*^+^] *ade1-14 his3Δ200:: HIS3::P_SUP35_SUP35R2E1 trp1-289 ura3-52 leu2-3, 112::LEU2::P_SUP35_NM(R2E1)-3HA SUP35R2E1* | SB787, SB1042 | This study |
| **SY3159** | *MATa* [*PSI*^+^] *ade1-14 his3Δ200 trp1-289 ura3-52, leu2-3, 112::LEU2::P_SUP35_NM-3HA-C* | SB1090 | This study |
| **SY3164** | *MATa* [*PSI*^+^] *ade1-14 his3Δ200 trp1-289 ura3-52 leu2-3, 112::LEU2:: ::P_SUP35_NM(ΔRPR)-3HA-C SUP35ΔRPR* | SB777, SB1092 | This study |
